# Ecological Repellent Preparations Based on Natural Polymers with the Addition of Essential Oils Acting on Ticks

**DOI:** 10.3390/insects15120931

**Published:** 2024-11-27

**Authors:** Monika Owczarek, Maria Wiśniewska-Wrona, Katarzyna Bartosik, Alicja Buczek, Monika Sikora, Klaudia Piekarska, Piotr Cichacz, Patryk Śniarowski, Zdzisława Mrozińska, Marcin H. Kudzin, Karolina Gzyra-Jagieła, Jagoda Jóźwik-Pruska

**Affiliations:** 1 Łukasiewicz Research Network–Lodz Institute of Technology, Skłodowskiej-Curie 19/27 St., 90-570 Łódź, Poland; maria.wisniewska-wrona@lit.lukasiewicz.gov.pl (M.W.-W.); monika.sikora@lit.lukasiewicz.gov.pl (M.S.); klaudia.piekarska@lit.lukasiewicz.gov.pl (K.P.); piotr.cichacz@lit.lukasiewicz.gov.pl (P.C.); patryk.sniarowski@lit.lukasiewicz.gov.pl (P.Ś.); zdzislawa.mrozinska@lit.lukasiewicz.gov.pl (Z.M.); marcin.kudzin@lit.lukasiewicz.gov.pl (M.H.K.); karolina.gzyra-jagiela@lit.lukasiewicz.gov.pl (K.G.-J.); jagoda.jozwik-pruska@lit.lukasiewicz.gov.pl (J.J.-P.); 2 Department of Biology and Parasitology, Faculty of Health Sciences, Medical University of Lublin, Radziwiłłowska 11 St., 20-080 Lublin, Poland; katarzyna.bartosik@umlub.pl (K.B.); alicja.buczek@umlub.pl (A.B.); 3 Institute of Materials Science and Engineering, Faculty of Mechanical Engineering, Lodz University of Technology, Żeromskiego 116 St., 90-924 Łódź, Poland; 4 Textile Institute, Faculty of Material Technologies and Textile Design, Lodz University of Technology, Żeromskiego 116 St., 90-924 Łódź, Poland

**Keywords:** ticks, *Dermacentor reticulatus*, *Ixodes ricinus*, repellent preparation, emulsions, biopolymers, essential oils, ecology

## Abstract

Background: Ticks (Acari: Ixodida) pose a serious medical and veterinary threat as vectors of tick-borne pathogens. The wide variety of tick repellents available on the market primarily consist of synthetic preparations that may disrupt the ecological balance and accumulate in the environment, leading to harmful effects on humans and animals. The aim of the study was to develop an ecological preparation based on natural raw materials (biopolymers) with the addition of a mixture of essential oils that act as tick repellents. Methods: The preparations were acquired through the emulsification method, specifically the oil-in-water emulsion technique. The assessment encompassed an analysis of their physicochemical properties, including centrifugal and thermal stability, dynamic viscosity, wetting angle, and conductivity. Additionally, their biodegradation and ecotoxicity profiles were evaluated, as well as their impact on tick behavior. Results: The preparations exhibited stability, rapid biodegradation, and absence of ecotoxicity. Additionally, they had repellent properties against the two tested species of ticks (*Ixodes ricinus* and *Dermacentor reticulatus*). Conclusions: Emulsion formulations comprising natural ingredients have significant research potential for combating ticks, thus mitigating the risk of tick-borne diseases in both human and animal populations.

## Simple Summary

A significant problem is the increasing incidence of tick-borne diseases. Ticks can be found in green areas, such as parks and clearings where trees, bushes, or tall grass grow. Humans and animals can become infected with various diseases through ticks, e.g., Lyme disease, tick-borne encephalitis, babesiosis, and anaplasmosis. An important aspect of preventing tick-borne diseases is the use of repellents to limit contact between ticks and humans and animals. Our research team has developed preparations based on natural raw materials with the addition of essential oils that act as a repellent against ticks. In addition to their pleasant scent, our preparations are environmentally friendly because they consist of natural ingredients.

## 1. Introduction

Ticks (Ixodida) are hematophagous parasites that infest terrestrial vertebrates and are prevalent across various climatic regions. These arthropods serve as significant vectors for a diverse array of pathogens, including, e.g., tick-borne encephalitis viruses, *Borrelia burgdorferi sensu lato* bacteria, *Anaplasma phagocytophilum*, *Ehrlichia* spp., spotted-fever group *Rickettsia* spp., *Francisella tularensis*, and *Babesia* spp. protozoa. As such, ticks are recognized as crucial vectors of infectious diseases affecting both humans and animals on a global scale [1].

The most significant tick-borne pathogen for public health is *Borrelia burgdorferi* s.l. spirochetes, which cause Lyme borreliosis, a multiorgan disease found on different continents. Over the past two decades, there has been a dramatic increase in the number of human infections with these pathogens in the USA and Europe [2,3].

Tick infestations not only cause local skin lesions and systemic reactions in both humans and animals [4,5,6,7,8], but they can also lead to tick paralysis [9,10,11] and trigger meat allergy, known as alpha-gal syndrome [12,13].

The importance of ticks in medicine and epidemiology, as well as the economic losses caused by their parasitic behavior on humans and animals, have led to increased interest in developing methods to protect potential hosts from tick attacks. It is advisable to search for effective ways to shield humans and animals from ticks, especially considering the expanding range and population of ticks, as well as their prolonged seasonal activity due to global warming and environmental changes [14,15,16].

To minimize the impact of tick parasitism, it is essential to shield hosts from tick attacks using a variety of anti-tick chemicals. Chemicals used in tick control encompass a wide range of options, including organochlorine compounds, formamidines, pyrethroids, organophosphates, phenylpyrazoles, carbamates, macrocyclic lactones, and more recently, spinosyns and isoxazolines [17,18]. Moreover, employing preventive measures such as repellents can serve as an additional layer of defense [19,20,21,22].

Ticks possess various biological features that contribute to their resilience and make them challenging to control. These include their high reproductive rate, allowing them to rapidly multiply in number. Additionally, ticks have the ability to travel long distances on animals, aiding in their widespread distribution. Their remarkable adaptability to different habitat conditions further complicates control efforts. Moreover, ticks have developed resistance to anti-tick chemicals, diminishing the effectiveness of chemical control methods in the long term [23,24]. Moreover, acaricides may be toxic to other organisms present in the environment [25,26,27,28]. Another limitation of the application of chemical compounds in tick control is the possibility of the development of tick resistance to various chemicals [29,30,31].

Due to these factors, there is a growing global trend towards the utilization of tick repellents and other measures to protect humans and animals from tick infestations [32,33,34,35,36]. This encourages the search for novel, effective products with repellent effects on various species of ticks with different forms of application.

The literature widely describes studies on both synthetic and natural repellents. The latter constitutes an attractive perspective for the use of repellents that are safe for the environment, biocompatible, and biodegradable [17]. The safe and effective control of tick populations is currently crucial, not only for human and animal health but also for agricultural and veterinary productivity. Overuse of previously employed repellents, including synthetic ones, has resulted in the development of resistance among most tick species [24]. In recent years, the interest in the use of plant substances to repel and/or kill ticks has been growing worldwide [33,37,38,39,40,41]. As reported by Benelli et al. [42], the majority of plants investigated for determination of their killing and repellent effects represent the families Asteraceae (15% of the selected studies), Fabaceae (9%), Lamiaceae (10%), Meliaceae (5%), Solanaceae (6%), and Verbenaceae (5%). In addition to the physicochemical properties and the active substance dose, the effectiveness of a repellent is influenced by various factors, such as the formulation type, e.g., cream, lotion, spray, or aerosol [43,44].

Among preparations based on essential oils, formulations in the form of micro- and nano-emulsions are very popular. This method of preparation enhances the solubility and bioavailability of the repellent. The preparation involves dispersing the essential oil phase in the water phase with the use of a surfactant to ensure emulsion stability. The promising results showing positive effects of essential oils from various parts of plants identified so far support the utilization of natural ingredients [45,46].

The objective of this study was to develop emulsion preparations using natural polymers (chitosan, cellulose derivative) and selected natural essential oils with potential anti-tick properties. The emulsions were characterized for their physicochemical, rheological, biodegradability, and ecotoxicity properties. Furthermore, the study examined the repellent effect of the formulations on two species of three-host ticks (*Ixodes ricinus* and *Dermacentor reticulatus*), which are widely distributed in Europe and have high epidemiological importance. Both these tick species infest companion animals and humans in their habitats.

## 2. Materials and Methods

### 2.1. Production of Emulsion Preparations

To prepare the oil-in-water emulsion, a 1% (*w*/*w*) aqueous solution of sodium carboxymethyl cellulose (CMC)—(250 kDa, Merck, Darmstadt, Germany) and a 1% (*w*/*w*) aqueous solution of chitosan (ChitoClear TM 4293, 235 kDa, DD = 86.8%, Primex EHF, Siglufjordur, Iceland) in 0.5% lactic acid (CHIT)—(p.a., 88%, Avantor, Gliwice, Poland) were used as the polymer base. Tween 80—*polyoxyethylene sorbitan monooleate* (Chempur, Piekary Śląskie, Poland) and SLP—*sorbitan laurate* + *polyglyceryl-4 laurate* + *dilauryl citrate* (Evonik Industries AG, Essen, Germany) were used as surface-active agents in a weight ratio of 1:1. Mixtures of essential oils (Naturalne Aromaty, Bochnia, Poland)—in equal parts of each essential oil, at 1% *w*/*w* of the total emulsion preparation, with potential repellent effects on ticks—were mixed with surfactants until a homogeneous mixture was obtained and then dripped into the polymer base with continuous stirring.

Ten emulsion preparations were developed with the compositions shown in Table 1.

The emulsions were submitted for further tests (biodegradation, ecotoxicity, physicochemical, rheological, and biological).

The GC-MS chromatographic analysis of all studied essential oils, including the mass spectra of the main compounds, is provided in Supplementary Material File S1.

### 2.2. Biodegradation and Ecotoxicity Tests of Preparations

Biodegradation tests were conducted according to international standard OECD 301B, which allows direct evaluation of the decomposition of material. The tested material (cream) was introduced to an inoculum in a closed environment in aerobic conditions. The minimum microbiological activity of the medium was 10^6^ cfu (colony-forming units). The biodegradability was determined by evaluating the production of CO_2_ over a minimum of 28 days in a liquid environment. The OECD 301 B test method is appropriate for materials that are highly soluble and poorly soluble, as well as for materials with certain concentrations recognized as insoluble. The method can be applied for fuels, essential oils, lubricants, surfactants, and personal care products.

The method for the classification of products was established under the terms of Ready or Ultimate Biodegradability. A material can be considered readily biodegradable if at least 60% of the organic carbon present in the material is converted to CO_2_ within a 10-day window and within 28 days in total, and ultimately biodegradable if 60% of the organic carbon in the material is converted to CO_2_ over the duration of the test [46].

In order to demonstrate the impact of the tested emulsions on the total number of microorganisms in the soil medium, microbiological tests were performed using the subculture method. The research was carried out in accordance with the accredited research procedure “Assessment of the influence of natural and synthetic materials on soil microflora”, developed on the basis of international standards [47,48,49,50]). Unfertilized universal soil was placed in the research reactors. The tested emulsions were sprayed on its surface, and then the samples were incubated for 28 days at a temperature of 30 °C. The initial soil was the reference sample (“0”). The tests were carried out using PCA agar medium. The total number of microorganisms in all reactors was monitored at specific time intervals.

### 2.3. Analytical Methods

#### 2.3.1. Conductivity of Preparations

Conductivity is determined by measuring the concentration of dissolved ions in the solution. This parameter is inversely proportional to resistivity. The specific electrical conductivity was analyzed using an EC 214 conductivity meter from HANNA Instruments (HANNA Instruments, Smithfield, George Washington Hwy, Chesapeake, VA, USA) at 20 ± 0.5 °C. The samples were analyzed after the manufacturing process and then after 4 and 8 weeks of storage at room temperature without exposure to light.

#### 2.3.2. Determination of the Dynamic Viscosity of Preparations Using the Brookfield Method

Dynamic viscosity [cP] of selected preparations was determined on a Brookfield model DV-II Pro digital viscosity meter equipped with Rheocalc V3.1-1 Software (Brookfield Engineering Laboratories, Middleborough, MA, USA) at 25 ± 0.1 °C. The CPE–40 cone for a volume of 0.5 cm^3^ was used for the measurement. The sample thermostating time was approximately 15 min. The measurement was performed according to the standard procedure developed at Łukasiewicz–ŁIT [51].

#### 2.3.3. Determination of the Stability of Preparations Using the Centrifugal Method

The centrifugal method was used to assess the stability of the emulsion preparations using a CentriFuge type MPW-340 (Mechanika Precyzyjna, Warsaw, Poland). Centrifugal testing of the preparations was carried out using centrifugal force. This test consisted of placing the tested emulsion systems in glass cuvettes and subjecting them to centrifugation at 4000 rpm for 10, 20, and 30 min [52].

#### 2.3.4. Determination of the Stability of Preparations with the Thermal Method

The thermal method was used to assess the stability of the preparations. The tested samples were kept at 25 ± 1 °C and humidity—29 ± 1% for 4 and 8 weeks. Based on an organoleptic evaluation, which was carried out weekly, changes in the macroscopic structure of the emulsion preparations were observed [53,54,55].

### 2.4. Wetting Angle Analysis of Preparations

The wetting angle analysis of the emulsion preparations was carried out on a Rame-Hart Model 90 goniometer using DROPImage Pro software Version 3.19.12.0 (Rame-Hart, Succasunna, NJ, USA). The measurement was made using the produced samples in contact with a glass plate. Five repeats were conducted for each measurement, and the arithmetic mean of the results was drawn.

### 2.5. Biological Tests on Ticks

#### 2.5.1. Ticks

Adult *I. ricinus* and *D. reticulatus* ticks (females and males) were collected in their habitats near Lublin (Eastern Poland, 51°14′53″ N 22°34′13″ E) during the spring peak of activity of both tick species (i.e., April–May 2024). The ticks were collected with the flagging method used commonly worldwide [56]. The specimens were placed in polypropylene vials and transported to the laboratory, where they were identified to the species using the identification keys elaborated by Nowak-Chmura [57].

Before the experiments, the ticks were kept in a refrigerator at a temperature of approx. 4 °C and approx. 80–95% RH in complete darkness for no longer than 48 h. Prior to the tests, the ticks had been adapted to the experimental abiotic conditions (20 ± 2 °C, approx. 50 ± 5% RH). The tests were conducted on only morphologically normal females and males with normal locomotory abilities.

In total, 392 ticks were used in the experiments, including 312 *D. reticulatus* adult specimens (208 females and 104 males) and 80 *I. ricinus* females. The inclusion of *D. reticulatus* females and males is justified by the fact that specimens of both sexes of this species attack humans and animals. In turn, in the case of *I. ricinus*, the behavior of females only was studied, as they most frequently attack and feed on host skin. Males of this species rarely attach themselves to the host or remain on host skin for a very short time.

#### 2.5.2. Study Procedure

To assess the repellent effect of the substance, an original method filed with the Patent Office of the Republic of Poland (application no. P.446360 [WIPO ST 10/C PL446360]) was employed. The method was developed based on multiyear observations of tick behavior in laboratory and field conditions. It is based on the natural traits of negative geotropism exhibited by all ticks in all their developmental stages (larvae, nymphs, and adults). When a repellent substance is used, ticks move away from its application site. The climbing tick assay used in this study involved observation of the tick behavior inside a vertically oriented open-top polypropylene tube with a marked scale in cm. To prevent ticks from escaping from the test tube, its opening was protected with a fabric (100% cotton with a weight of 210 g/m^2^) where the tested emulsions were applied. A mixture that prevented the ticks from moving at a distance of at least 3 cm from the source of the tested emulsion was considered to have a repellent effect. Simultaneously, the control group, in which the same amount of H_2_O was used instead of the tested substance, was established. The longer the distance from the site where the tick remained, the stronger the repellent effect of the tested substance. In the methodology used in the study, the effect of the tested substance was estimated based on the distance at which the ticks were found from the source of the potential repellent at 15, 30, 45, 60, 90, 120, 150, 180, and 240 min after the application.

To assess the repellent properties of the tested emulsions, the criteria for assessment of the repellent efficacy of the products specified in the European Union and the USA were adopted [58]. According to the EU directives, a substance that repels at least 90% of tick specimens per time unit is considered effective. In the US Agency recommendations, the number of repelled specimens per time unit should be greater than/equal to 95% of all tested specimens.

The following analyses were performed to assess the effectiveness of the 10 tested emulsions with a potential repellent effect on ticks:

- Analysis of the behavior of *D. reticulatus* females and males under the influence of the substances applied at the doses of 250 µL/7 cm^2^ and 500 µL/7 cm^2^ in the test area;
- Analysis of the behavior of *D. reticulatus* females and males under the influence of the carrier/base/matrix substances used in the tested formulations, i.e., CMC and CHIT applied in the test at the doses of 250 µL/7 cm^2^ and 500 µL/7 cm^2^;
- Analysis of the behavior of *I. ricinus* females under the influence of the substances applied at the dose of 250 µL/7 cm^2^. Emulsions CMC 2, CMC 3, CHIT 2, and CHIT 4, which exerted a clear repellent effect on the *D. reticulatus* ticks, were selected for this analysis.

#### 2.5.3. Statistical Analysis

The Z-test for two independent proportions with continuity correction was applied to compare the percentages of ticks exposed to the matrices used in the tested emulsions (CMC and CHIT). Two hypotheses were examined: a two-sided *p*-value, which indicates whether there is a difference in the proportions (percentages of ticks) between the matrix-exposed and control ticks, and a one-sided *p*-value to determine whether the proportion (percentage of ticks) for the matrix exposure is greater than the proportion (percentage of ticks) for the control. A *p* value of <0.05 was considered statistically significant. The statistical calculations were carried out using the PQStat Software statistical package version 1.4 (Poznań, Polska).

The repellency percentage was calculated for the studied emulsions at 15, 30, 45, 60, 90, 120, 150, 180, and 240 min post application according to the Henderson equation/Modified Abott’s formula:

$$100-\frac{mean\;number\;of\;ticks\;not\;repelled}{mean\;number\;of\;ticks\;not\;repelled\;on\;controls}\times 100$$

## 3. Results

### 3.1. Production of Emulsion Preparations

Oil-in-water emulsions were prepared using aqueous solutions of the biopolymers (CMC or CHIT) combined with a selected blend of essential oils. These emulsions were formed by intensive mixing in the presence of surfactants SLP and Tween 80. All the resulting preparations had a creamy consistency and a milky color and emitted a pleasant fragrance.

### 3.2. Biodegradation and Ecotoxicity Tests

**Figure 1:**
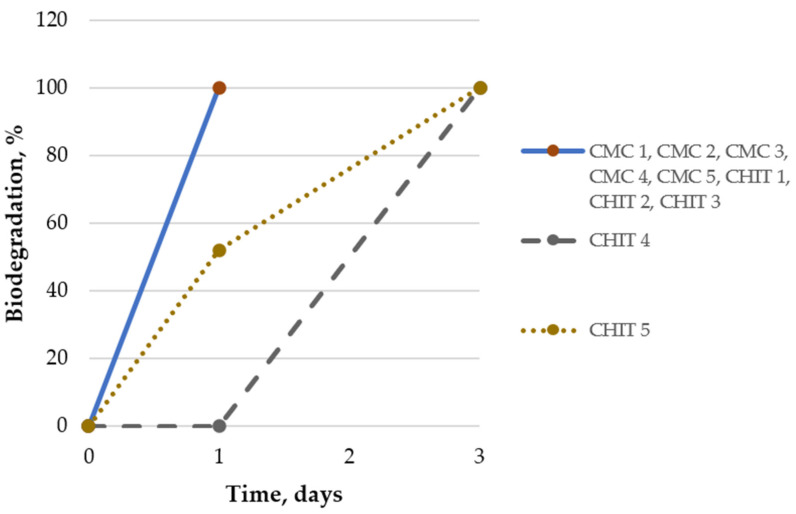
Biodegradation rate of emulsion preparations.

The tests showed that the emulsions were susceptible to biodegradation (100%). The decomposition time of the samples in the water environment was maximum 3 days. According to the OECD 301 B guidelines and based on the obtained results, it can be concluded that the designed product is rapidly biodegradable. Figure 1 illustrates the biodegradation process.

The examination of the impact of the composition and degradation of the tested emulsions on the total number of microorganisms in the soil medium showed no significant effect. The total number of microorganisms in the soil on experiment day 0 was 1.1 × 10^6^ cfu (*colony-forming units*); next, it decreased and remained at a constant level of 10^5^ cfu in all the tested samples.

**Figure 2:**
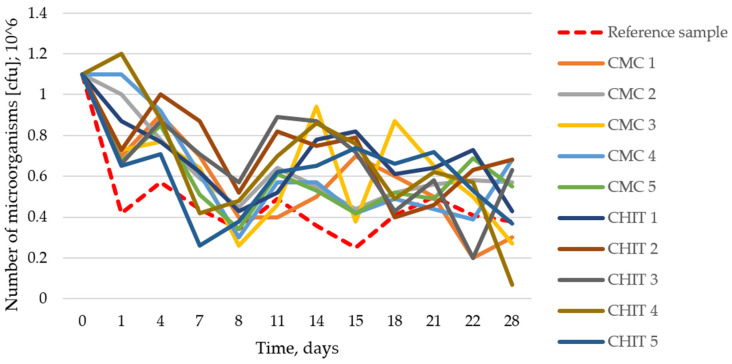
Influence of the composition and degradation of emulsions on the total number of microorganisms in the soil medium; reference sample—initial soil.

Table 2 and Figure 2 show the results of the impact of the tested emulsions on the total number of microorganisms in the soil medium.

### 3.3. Analytical Results

#### 3.3.1. Conductivity of Preparations

**Figure 3:**
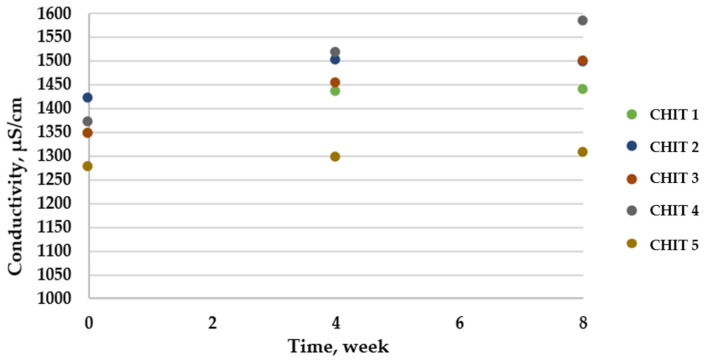
Conductivity of chitosan-based emulsion preparations.

**Figure 4:**
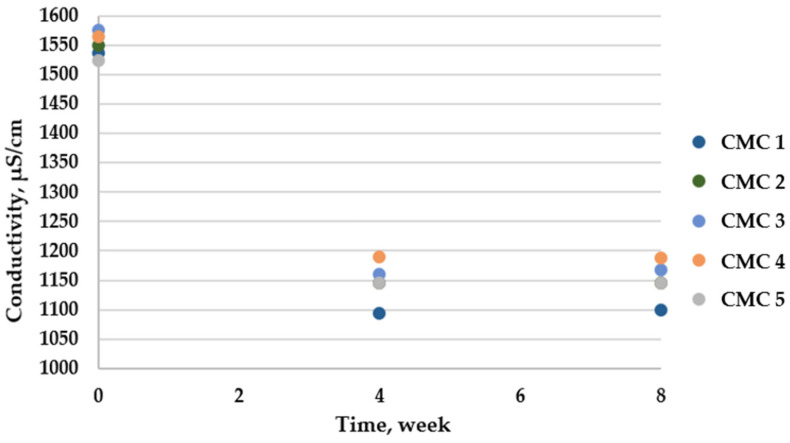
Conductivity of CMC-based emulsion preparations.

The data show that the conductivity values decreased over time for the CMC samples but increased for the CHIT samples (Figure 3 and Figure 4). The ability of solutions to conduct electricity is related to ion migration. Therefore, measuring conductivity can provide information about the number of ions in the solution. As polymers degrade, new chemical structures may form, leading to an increase in conductivity values. The resulting degradation products increase the number of ions, thus increasing conductivity. This effect is observed for easily degradable biopolymers like chitosan. For each CHIT sample, the additives in the emulsion influenced the conductivity values at time zero. The change in the conductivity values after 4 weeks of storage averaged around 6.5%, and after 8 weeks, the conductivity values stabilized (Figure 3). CHIT 5 was the most stable chitosan-based sample.

The CMC-containing samples showed the most significant differences in the studied parameter. Unlike the other biopolymers, there was a decrease in the value over time, but the additives did not have a significant effect on the emulsion value at time zero. In the case of this emulsion matrix, the absorption of emulsion components and the formation of linkages may have occurred, resulting in a reduction in the ion concentration in the solutions. Over a span of 4 weeks, a 26% decrease in the average value was observed due to storage, and stabilization occurred after 8 weeks (Figure 4). CMC 5 was the most stable sample.

#### 3.3.2. Dynamic Viscosity (Brookfield Method) of Preparations

**Figure 5:**
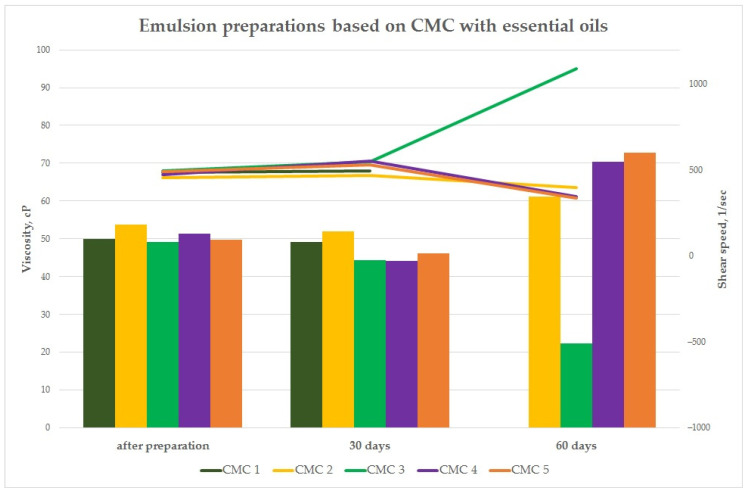
Dynamic viscosity of emulsion preparations based on CMC at specified intervals.

**Figure 6:**
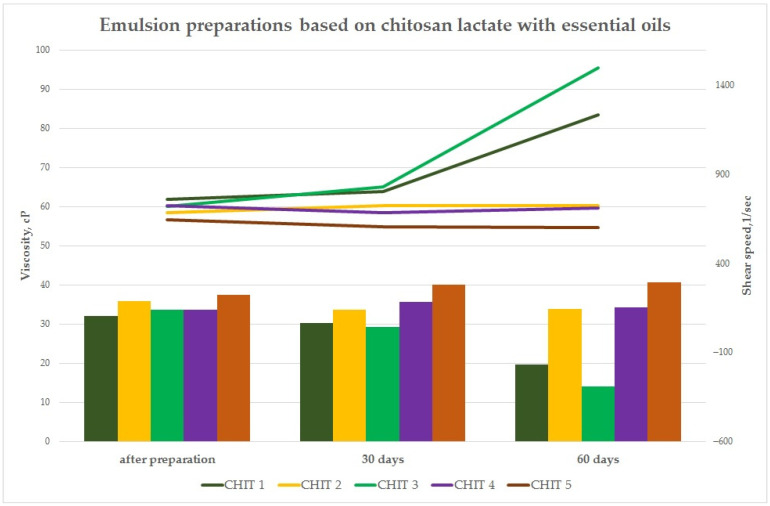
Dynamic viscosity of emulsion preparations based on CHIT at specified intervals.

As part of the study, the rheological properties of the emulsion preparations with the essential oil mixture were evaluated immediately after production and then at specified intervals (30 days and 60 days). The purpose of these studies was to assess the effect of the essential oil mixtures on the viscosity of the preparations. This is extremely important from the point of view of developing the final form of the preparation for application to surfaces in green areas. The study was performed using a Brookfield model DV-II digital viscosity meter equipped with the Rheocalc V3.1-1 Program at 25 ± 0.1 °C. The CPE-40 cone was used for the measurement of a volume of 0.5 mL. The results from this stage of the study are shown in the graphs (Figure 5 and Figure 6).

In the case of the CMC-based preparations, a decrease was observed at the levels of 54.54 cP for the CMC 3 sample. In the case of preparations CMC 2, CMC 4, and CMC 5, the dynamic viscosity increased after 60 days to the levels of 61.11, 70.35, and 72.70 cP, respectively (Figure 5). A significant decrease in dynamic viscosity was observed for some emulsion preparations based on chitosan lactate after a period of 60 days. The decrease in this parameter was at the level of 38.72 cP for the CHIT 1 sample and 56.65 cP for the CHIT 3 sample (Figure 6).

#### 3.3.3. Stability of Emulsion Preparations Assessed with Centrifugation and Thermal Methods

The primary methods for assessing the stability of emulsion preparations are centrifuge and thermal tests carried out immediately after production and after a suitable period of time in certain conditions. Complex methods of stability assessment use the phenomenon of multiple light scattering and laser diffraction. Determination of the effect of ambient conditions on the stability of preparations is possible during storage thereof in ambient conditions or in appropriate incubators.

One of the basic methods for assessing the stability of emulsion preparations is centrifugal testing using centrifugal force. This test involves placing the tested emulsions in glass cuvettes and subjecting them to the centrifugation process. Rotation causes the unstable preparations to stratify into visible aqueous and essential oil phases.

The stability of the emulsion preparations was evaluated 24 h after they were manufactured. The results of the stability of the emulsion preparations subjected to centrifugation are shown in Table 3.

The tests showed that, within the specified time intervals (10, 20, and 30 min) at 4000 rpm, all the emulsions (based on CMC and CHIT) with the addition of essential oils did not separate and remained stable. The results of the stability of the emulsion preparations assessed with the thermal method are shown in Table 4.

The results of the organoleptic assessment (Table 4) showed that, after 30 days of storage at a temperature of 25 ± 1 °C and humidity of 29 ± 1%, all the emulsion preparations (based on CMC and CHIT) with the essential oil mixtures did not separate and remained stable. However, after 60 days in these conditions, separation (sedimentation process) of some preparations was observed. It is a reversible process involving the settling of particles in a liquid under the influence of gravitational or inertial forces. It does not change the properties of the preparations, which only need to be mixed thoroughly before use.

### 3.4. Wetting Angle Analysis of Preparations

**Figure 7:**
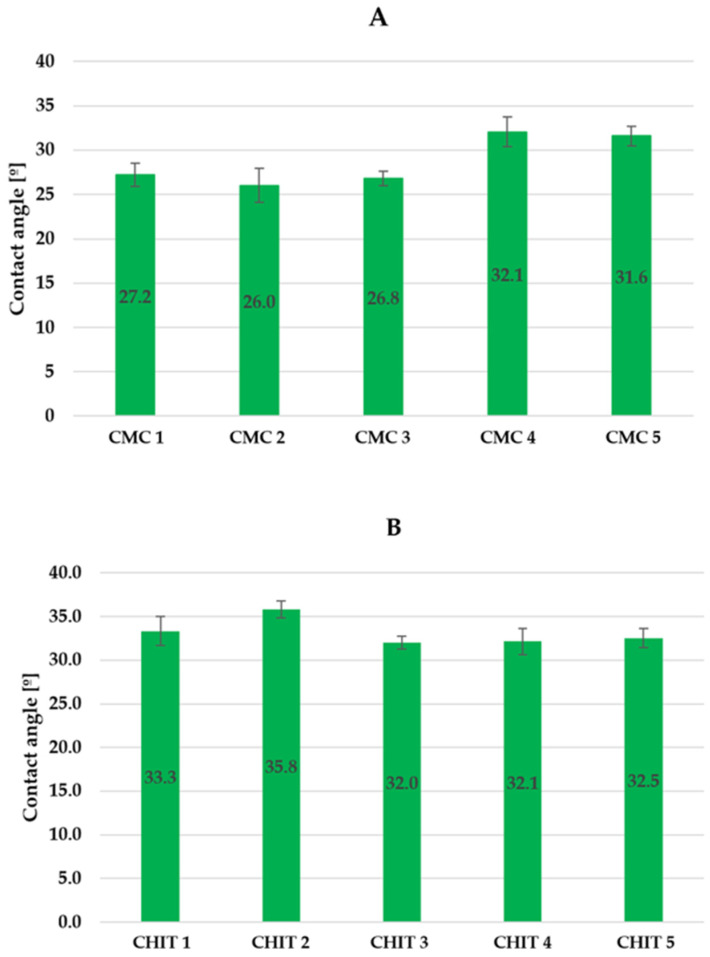
Contact angle of CMC emulsions (**A**) and CHIT emulsions (**B**) on the glass plate.

The results of the wetting angle analysis with the standard deviations are summarized in Figure 7A,B. The values obtained for both types of emulsions were very similar. The CMC emulsions had wetting angle values ranging from 27.2° to 32.1°, whereas the results for the preparations from the CHIT group ranged from 32.0° to 35.8°.

**Figure 8:**
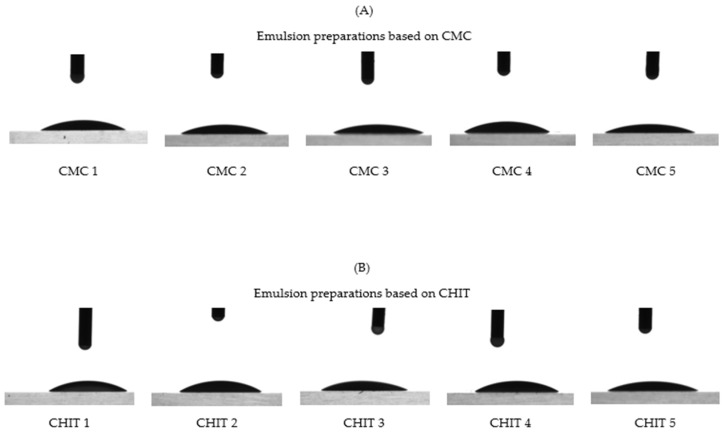
Contact angle of CMC emulsions (**A**) and CHIT emulsions (**B**).

The behavior of the preparations in contact with the glass plates is shown below in Figure 8.

### 3.5. Tests on Ticks

#### 3.5.1. Effect of Tested Substances on *Dermacentor reticulatus* Ticks

**Figure 9:**
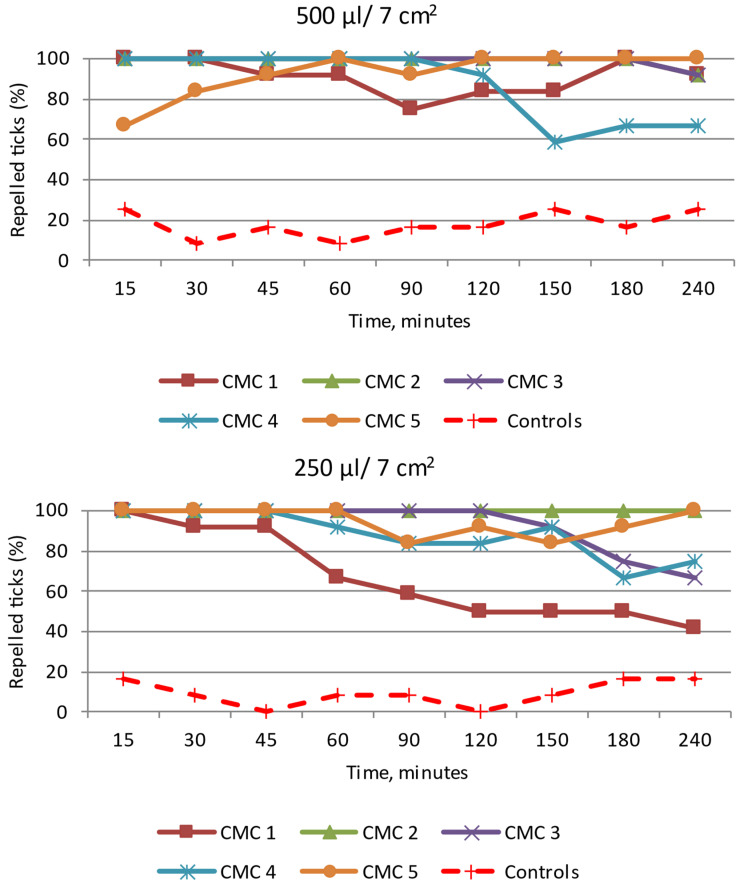
Behavior of *Dermacentor reticulatus* adults under the influence of selected emulsion preparations based on CMC, e.g., CMC 1—CMC 5; repelled ticks (%)—specimens present at a distance greater than/equal to 3 cm from the source of the tested emulsion. In every experimental group, as in the control, 12 adult ticks (8 females and 4 males) were used.

CMC 1: The analysis of the effect of the application of this emulsion at the doses 500 µL/7 cm^2^ and 250 µL/7 cm^2^ showed that it did not meet the requirements set for repellents. After the application of 500 µL/7 cm^2^ of the emulsion, 100% of females, males, and *D. reticulatus* adults in total were present at a distance of ≥3 cm from the repellent source for only 60 min, 30 min, and 30 min, respectively (Figure 9 and Table S1). The majority of ticks from the control group were found close to the water application site. The calculations made using the Henderson equation/Modified Abott’s formula confirmed that the repellency percentage was 100% for 30 min at the dose of 500 µL/7 cm^2^ and for 15 min at the dose of 250 µL/7 cm^2^. At these periods, the tick distribution in the tested zones differed significantly between CMC 1 and the control for both doses tested, with Z ranging from 3.37 to 4.10; *p* < 0.05 and Z = 3.73; *p* < 0.05, respectively.

CMC 2: The readings at all the time points revealed a strong repellent effect of this emulsion against *D. reticulatus* ticks after the application of 500 µL/7 cm^2^ and 250 µL/7 cm^2^ (Figure 9 and Table S2). A total of 100% of females and males did not move closer than 3 cm from its source. Only one male was found close to the repellent source after 240 min. In the control group, the majority of the ticks were present within the zone 3 cm away from the water application site in both these experiments (Table S2). The repellency percentage was 100% for 180 min at the dose of 500 µL/7 cm^2^ and for 240 min at the dose of 250 µL/7 cm^2^. At these time points, the tick distribution in the tested zones differed significantly between CMC 2 and the control for both doses tested, with Z ranging from 2.90 to 4.10; *p* < 0.05 and Z ranging from 3.73 to 4.49; *p* < 0.05, respectively.

CMC 3: After the application of 500 µL of the emulsion/7 cm^2^, the consecutive readings at the time points from 15 min to 180 min after the beginning of the tests showed that none of the specimens of both sexes approached the source of the stimulus at a distance less than 3 cm (Figure 9 and Table S3). After 240 min, only one *D. reticulatus* female was found in the area of emulsion 3 application. A slightly weaker repellent effect was observed after the application of 250 µL/7 cm^2^ of the emulsion. A total of 100% of adult ticks were present at a distance greater than/equal to 3 cm from the repellent source for 120 min. The repellency percentage was 100% for 180 min at the dose of 500 µL/7 cm^2^ and for 120 min at the dose of 250 µL/7 cm^2^. At these time points, the tick distribution in the tested zones differed significantly between CMC 3 and the control for both doses tested, with Z ranging from 3.73 to 4.10; *p* < 0.05 and Z ranging from 3.73 to 4.49; *p* < 0.05, respectively.

CMC 4: After the application of 500 µL/7 cm^2^ of the emulsion, 100% of the *D. reticulatus* specimens (females, males, and adults in total) were present at a distance greater than/equal to 3 cm from the source of the substance at the time points from 15 min to 90 min (Figure 9 and Table S4). After this time, some specimens were found at the site of application of this emulsion, which suggests no repellent effect of this formulation. After the use of 250 µL of the emulsion, the ticks were present at the application site already after 60 min (Table S4). The calculations carried out using the Henderson equation/Modified Abott’s formula confirmed that the repellency percentage was 100% for 90 min at the dose of 500 µL/7 cm^2^ and for 45 min at the dose of 250 µL/7 cm^2^. At these time points, the tick distribution in the tested zones differed significantly between CMC 4 and the control for both doses tested, with Z ranging from 3.37 to 4.10; *p* < 0.05 and Z ranging from 3.73 to 4.49; *p* < 0.05, respectively.

CMC 5: The experiment results indicated a low repellent effect of this emulsion used in both groups (Figure 9 and Table S5). The repellency percentage of 100% was observed and calculated only at the dose of 250 µL/7 cm^2^ for 60 min. At that time, the tick distribution in the tested zones differed significantly between CMC 5 and the control (Z ranging from 3.37 to 4.49; *p* < 0.05).

**Figure 10:**
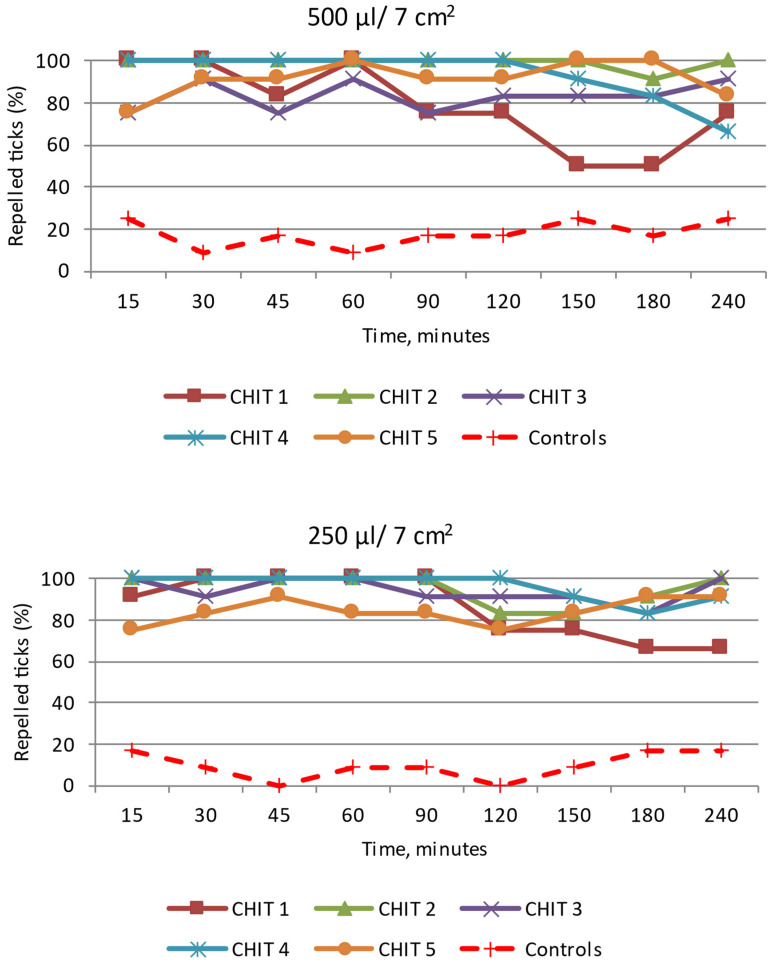
Behavior of *Dermacentor reticulatus* adults under the influence of selected emulsion preparations based on CHIT, e.g., CHIT 1—CHIT 5; repelled ticks (%)—specimens present at a distance greater than/equal to 3 cm from the source of the tested emulsion. In every experimental group, as in the control, 12 adult ticks (8 females and 4 males) were used.

CHIT 1: Although the tested emulsion exerted a 100% repellent effect for 30 min and 90 min after the application of 500 µL/7 cm^2^ and 250 µL/7 cm^2^ (Z ranging from 3.37 to 4.10; *p* < 0.05 and Z ranging from 3.73 to 4.49; *p* < 0.05, respectively), it does not fully meet the requirements set for effective repellents due to the short time of full protection (Figure 10 and Table S6).

CHIT 2: All the readings revealed a strong repellent effect of the emulsion on the *D. reticulatus* ticks after the application of 500 µL/7 cm^2^ (Figure 10 and Table S7). In the experiments consisting in the use of 250 µL of the mixture, 100% of the specimens were found at a distance greater than/equal to 3 cm from the source of the substance at the time points of 15, 30, 45, 60, and 90 min (Table S7). After 120, 150, and 180 min from the beginning of the experiments, single specimens moved towards the site of application of the emulsion but left the area soon. This behavior may indicate a repellent effect of this emulsion. The repellency percentage of 100% was observed at the dose of 500 µL/7 cm^2^ for 150 min and at the dose of 250 µL/7 cm^2^ for 90 min. At these time points, the tick distribution in the tested zones differed significantly between CHIT 2 and the control (Z ranging from 3.37 to 4.10; *p* < 0.05 and Z ranging from 3.73 to 4.49; *p* < 0.05, respectively).

CHIT 3: When exposed to this emulsion, the ticks moved over the entire surface in both experimental groups during the observation period (Figure 10 and Table S8). This indicates a weak repellent effect of the emulsion. Although the tick distribution in the tested zones differed significantly between CHIT 3 and the control (Z ranging from 2.04 to 3.67 *p* < 0.05 and Z ranging from 2.86 to 4.49 *p* < 0.05, respectively), no persistent repellent effect was observed at any of the doses of the tested emulsion (Table S8).

CHIT 4: After the application of 500 µL/7 cm^2^ of the emulsion, no specimens of both sexes approached the source of the chemical stimulus at a distance less than 3 cm at the time points from 15 min to 120 min (Figure 10 and Table S9). Similar results were obtained after the application of the smaller amount of the mixture (250 µL) to the 7 cm^2^ surface (Table S9). A 100% repellent effect was observed for 120 min at both doses of the tested emulsion, and the tick distribution in the tested zones differed significantly between CHIT 4 and the control (Z ranging from 3.73 to 4.10 *p* < 0.05 and Z ranging from 3.73 to 4.49 *p* < 0.05, respectively).

CHIT 5: In both experimental groups, the *D. reticulatus* ticks moved within the observation field (Figure 10 and Table S10). At each of the time points, the females were found at the site of application of this substance. These results indicate a weak repellent effect of this emulsion. Although the tick distribution in the tested zones differed significantly between CHIT 5 and the control (Z ranging from 2.04 to 3.67; *p* < 0.05 and Z ranging from 2.86 to 4.49; *p* < 0.05, respectively), a 100% repellency effect was not observed at any of the time points.

#### 3.5.2. Effect of Emulsions on *Ixodes ricinus* Females

**Figure 11:**
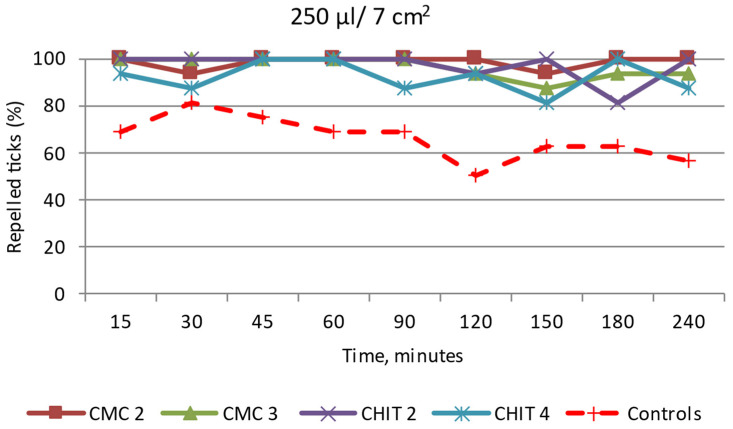
Behavior of *Ixodes ricinus* females under the influence of selected emulsion preparations based on CMC e.g., CMC 2 and CMC 3, and based on CHIT e.g., CHIT 1—CHIT 5; repelled ticks (%)—specimens present at a distance greater than/equal to 3 cm from the source of the tested emulsion. In the test groups, as in the control, 16 females of *I. ricinus* were used.

CMC 2: After the application of 250 µL/7 cm^2^ of the mixture, the *I. ricinus* females were present at a distance greater than/equal to 3 cm from the stimulus source at most of the time points (Figure 11 and Table S11). At two time points, single females approached the source of the emulsion but moved in the opposite direction shortly.

The tick distribution in the study period in the tested zones differed significantly between CMC 2 and the control (Z ranging from 1.60 to 1.95; *p* < 0.05), except for one time point, i.e., 30 min (Z = 1.21, *p* = 0.1126).

Since the tested ticks crossed the test field boundary twice, the calculations made using the Henderson equation/Modified Abott’s formula did not show a sustained 100% repellent effect. However, studies on the repellent effect of this emulsion with different concentrations should be continued.

CMC 3: At the time points from 15 min to 90 min from the beginning of the study, 100% of the *I. ricinus* females were found at a distance greater than/equal to 3 cm from the source of the substance (Figure 11 and Table S11). After 120 min, the females were present on the surface covered with the emulsion. The calculation showed that the substance exerted a 100% repellent effect for only 90 min after application. The tick distribution for this period in the tested zones differed significantly between CMC 3 and the control (Z ranging from 1.60 to 1.95; *p* < 0.05), except for one time point, i.e., 30 min (Z = 1.21, *p* = 0.1126).

CHIT 2: The *I. ricinus* females did not approach the surface covered with the emulsion at a distance of less than 3 cm at most of the time points (Figure 11 and Table S11). This indicated that the analyzed substance had a repellent effect throughout the entire observation period. However, a 100% repellent effect was observed only for 90 min, and the tick distribution in this period in the tested zones differed significantly between CHIT 2 and the control (Z ranging from 1.60 to 1.95; *p* < 0.05), except for one time point, i.e., 30 min (Z = 1.21, *p* = 0.1126).

CHIT 4: Throughout the experiment, the *I. ricinus* females moved within the entire observation area, including the emulsion application site (Figure 11 and Table S11). Based on these results, it can be concluded that the emulsion applied at the dose of 250 µL/7 cm^2^ does not meet the requirements set for repellents against *I. ricinus* ticks. The tick distribution between the tested zones did not differ significantly between the tested emulsion and the control in most of the time points (Z ranging from 0.00 to 1.60, *p* ≥ 0.05).

#### 3.5.3. Effect of the Biopolymer Base of the Emulsions: CMC (Sodium Carboxymethyl Cellulose) and CHIT (Chitosan Lactate) on *D. reticulatus*

**Figure 12:**
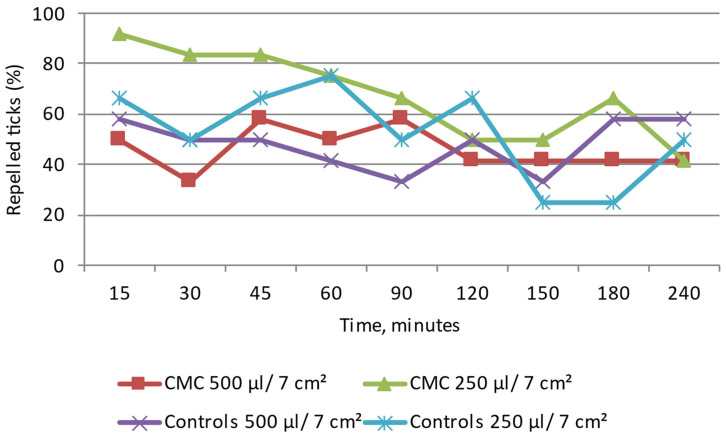
Behavior of *Dermacentor reticulatus* adults under the influence of CMC (polymer base of emulsion); repelled ticks (%)—specimens present at a distance greater than/equal to 3 cm from the source of the tested emulsion. In every experimental group, as in the control, 12 adult ticks (8 females and 4 males) were used.

CMC: Throughout the study, the *D. reticulatus* ticks moved in different directions. As in the control, after the application of both amounts of the emulsion to the 7 cm^2^ surface, numerous specimens were found at the application site at all the reading time points (Figure 12 and Table S12). At a distance greater than/equal to 3 cm from the site of application of 500 µL and 250 µL/7 cm^2^ of the emulsion, from 33.3% to 58.3% and from 50% to 91.6% of adult ticks were found, respectively. The tick distribution between the tested zones did not differ significantly between the sodium carboxymethyl cellulose matrix trials and the control (Z ranging from 0.41 to 1.64; *p* > 0.05). The present results indicate no repellent effect of this emulsion used in both experimental groups. The maximum value of the repellency percentage according to the Henderson equation/Modified Abott’s formula calculated for CHIT was 75.0%.

**Figure 13:**
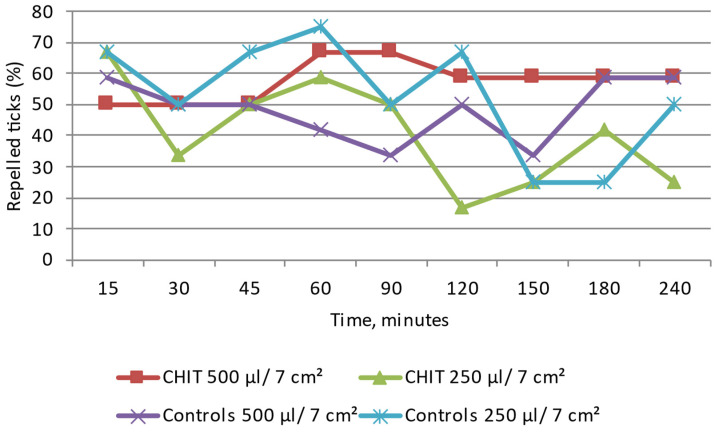
Behavior of *Dermacentor reticulatus* adults under the influence of CHIT (polymer base of emulsion); repelled ticks (%)—specimens present at a distance greater than/equal to 3 cm from the source of the tested emulsion. In every experimental group, as in the control, 12 adult ticks (8 females and 4 males) were used.

CHIT: Throughout the observation period, the *D. reticulatus* specimens of both sexes moved over the entire observation surface, and a large percentage of ticks were present at the site of application of 500 µL and 250 µL/7 cm^2^ of the emulsion (Figure 13 and Table S13). The behavior of the ticks in these tests was similar to that observed in the control group. As shown by the experiment, the emulsion does not have repellent activity. The tick distribution in the tested zones did not differ significantly between the chitosan lactate matrix trials and the control (Z ranging from −1.27 to 0.22; *p* > 0.05). The maximum value of the repellency percentage calculated for CMC was 22%.

Based on the analysis of the results of the experiments conducted on *D. reticulatus* and *I. ricinus* ticks, the 10 emulsions can be classified into three groups:

(1) The first group comprises emulsions CMC 2 and CHIT 2, which exert the strongest repellent effect on both tick species and provide protection against ticks in accordance with the criteria adopted in the EU and the US [58].
(2) The second group is represented by CMC 3 exhibiting repellent potential against both tick species, but its 100% repellent effect lasts for a shorter time, i.e., from 120 min to 180 min, depending on the tick species and/or the amount of the substance applied. The substance met only the EU requirements during this period [58].
(3) The third group comprises emulsions CMC 1, CMC 4, CMC 5, CHIT 1, CHIT 3, and CHIT 9, i.e., formulations with the weakest repellent effects that did not meet the EU and USA criteria for repellent effectiveness [59].

## 4. Discussion

Various synthetic substances and plant extracts are used to repel ticks and other blood-ingesting arthropods [42,60,61,62,63,64]. Four of these repellents, i.e., three synthetic substances—N,N-diethyl-3-methylbenzamide (DEET), (1-methyl-propyl-2-(hydroxyethyl)-1-piperidinecarboxyla-te (picaridin or KBR 3023), and 3-(N-acetyl-N-butyl) aminopropionic acid ethyl ester (IR 3535 or EBAAP)—and only one plant-based repellent, i.e., lemon-scented eucalyptus oil (p-menthane-3,8-diol), are recommended by the Centers for Disease Control and Prevention for personal protection of humans against arthropod infestations [65,66,67]. In recent years, there has been a growing preference for natural substances that protect against tick attacks. This preference is likely due to the perceived lower toxicity of natural substances and the documented reactions in humans caused by synthetic substances like DEET [68], KBR 3023 [69], and IR3535 [70]. Consequently, there has been increased interest in the investigation of the effects of plant extracts on tick behavior and in the development of repellents [37,71,72,73].

The repellent activity of essential oils and plant extracts has previously been confirmed in studies on *I. ricinus* nymphs [62,74,75,76,77,78,79,80,81]. In contrast, the effect of natural products on *D. reticulatus* ticks has been studied less frequently to date [64].

The emulsion preparations were formulated using aqueous biopolymer solutions containing dispersed essential oil droplets and surfactants (SLP and Tween 80). These preparations demonstrated stability for 60 days upon organoleptic evaluation. Studies indicate that these environmentally friendly formulations may have a repellent effect on ticks. Scientific reports have confirmed the biodegradability of CMC in diverse environments, including water and soil [82,83]. Additionally, CMC intermediates were found to have no toxic effects on *Selenastrum capricornutum* (algae), *Daphnia magna* (water flea), and *Brachydanio rerio* (zebra fish). Chitosan, another natural polymer, also exhibits high biodegradation potential in various environments [84,85]. Our study verified the biodegradability of both polymers in water environments and their non-harmful impact on microorganisms.

Essential oils are categorized as complex reaction products or biological materials (UVCBs). In accordance with the law in force in the USA and the European Union, importers and producers of chemicals are obliged to carry out a risk assessment of the chemicals they place on the market [86,87,88]. The standardized tests cannot be applied to UVCBs. There is limited literature on the biodegradability of essential oils. The stability and degradation of essential oils are linked to their potential to oxidize. External factors, such as temperature, light, metal contaminants, water content, compound structure and chemical composition, and exposure to atmospheric oxygen, influence this process. Many essential oils evaporate within 1–8 h [89].

The study demonstrated that the tested emulsion, consisting of a natural polymer matrix and essential oils, is highly biodegradable. It was observed that this composition did not have any harmful effects on soil microorganisms. The high susceptibility to decomposition may be attributed to the physical combination of the ingredients and the high vapor pressure of the oils.

The study of the rheological properties for the emulsion preparations based on aqueous solutions of biopolymers with essential oils showed that the value of dynamic viscosity [cP] was significantly influenced by the qualitative composition of the preparations, i.e., the polymer matrices used and the composition of the essential oil mixtures. Dynamic viscosity measurement is also important from the point of view of developing the final quantitative and qualitative composition of emulsion preparations. It also plays an important role in the proper selection of technological parameters of the equipment for the production of emulsion components and the final product, as well as applications in real conditions in green areas. The decrease in viscosity for some preparations (CMC 3, CHIT 1, and CHIT 3) may indicate hydrolytic degradation of the polymer matrix itself due to the breaking of chemical bonds in macromolecules under the influence of the aqueous environment, which is a natural process associated with aging of natural polymers of the polysaccharide group stored in the liquid form for a long time and the action of the essential oil mixture itself as a factor causing degradation of the chitosan and cellulose matrix. The studied emulsion systems exhibit the characteristics of non-Newtonian pseudoplastic (shear-thinning) liquids, for which the dynamic viscosity decreases with an increasing shear rate.

All the emulsions produced had very similar wetting angle values around 30°. This suggests that they possess a strong hydrophilic quality, which aids in their ability to spread across surfaces. In comparison, water achieves values of around 90° when in contact with glass, indicating its lower surface energy [90]. Currently, there are no existing studies in the literature on the wettability of surfaces by formulations with a repellent effect on ticks. Nevertheless, this study holds significance in evaluating the ease with which a substance can be distributed over surfaces, such as plants.

The results of the present study indicate that one of the five mixtures of plant essential oils, i.e., the mixture of lavender + eucalyptus + tea tree with two matrices—CMC and CHIT (emulsions CMC 2 and CHIT 2) used at 250 µL/7 cm^2^, exhibits high repellent efficacy against adults of both *D. reticulatus* and *I. ricinus* for at least 4 h after application. The finding of the strong repellent effect on both these tick species is highly important, as both species occur in the same habitats in urban and non-urban areas in some regions of Europe. Given the high prevalence of tick-borne pathogens vectored by ticks, these arthropods pose a serious threat to humans and animals present in tick habitats [91,92,93].

In the present study, the mixtures of the other essential oils exhibited weaker repellent activity against both tick species and did not meet the requirements set by the EU and the US for repellents used for protection against tick attacks [59].

Previously, Jaenson et al. [60] conducted laboratory tests and confirmed the repellency of lemon-scented eucalyptus, geranium, and lavender oils and the mosquito repellent MyggA Natural (composed of 30% of lemon-scented eucalyptus with a minimum of 50% p-menthane-3,8-diol and a smaller amount of lavender and geranium essential oils) against *I. ricinus* nymphs. The repellency of the tested essential oils at 2 and 5 min after the application was estimated at 100% (MyggA Natural), 30% (lemon-scented eucalyptus oil), and 30% (lavender oil). The duration of the repellent effect of the essential oils was not analyzed in the study. In a field study carried out using the blanket-dragging technique for 4 days during a 6 d period, the repellency of MyggA Natural and lemon-scented gum (*Corymbia citriodora*) oil ranged from 45.2 to 77.3% and from 41.6 to 85.2%, respectively [60]. PMD (cis- and trans-p menthane-3,8-diol), i.e., the main active ingredient of *C. citriodora* oil, applied on rabbit ears at a dose of 0.36 l/cm^2^, significantly reduced attachment and feeding of *I. ricinus* nymphs [94].

Still little is known about the repellent efficacy of natural substances against *D. reticulatus* ticks. As reported by Štefanidesová et al. [64], the greatest effectiveness in repelling these arthropods was exhibited by clove bud (*Syzygium aromaticum*), creeping thyme (*Thymus serpyllum*), and red thyme (*Thymus vulgaris*) oils, which repelled 83, 82, and 68% of tick specimens, respectively, when applied as a 3% dilution.

Various tick species react in different ways to the presence of chemical substances. For example, the formulation composed of thyme oil + peppermint oil with CMC exerted a stronger repellent effect on adult stages of *D. reticulatus* than *I. ricinus*. Similarly, nymphs of blacklegged ticks (*Ixodes scapularis*) have been shown to be less sensitive to repellents than lone star ticks (*Amblyomma americanum*) [95].

The high repellent activity of the tested CMC + lavender + eucalyptus + tea tree and CHIT + lavender + eucalyptus + tea tree emulsions against *D. reticulatus* and *I. ricinus* persisted longer than that of 15% DEET and 5% geraniol in laboratory two-choice bioassays conducted on *Amblyomma americanum*, *Dermacentor variabilis*, *Ixodes scapularis*, and *Rhipicephalus sanguineus* ticks [96].

In the present study, the effect of single essential oils was not analyzed; hence, it is impossible to determine whether the use of the mixture of three plant essential oils—lavender + eucalyptus + tea tree—with two matrices (CMC and CHIT) increases the repellent efficacy of the emulsion and has an impact on the persistence of the high activity against *D. reticulatus* and *I. ricinus* adults. As reported by Štefanidesová et al. [64], a mixture of creeping thyme (*Thymus serpyllum*) and citronella (*Cymbopogon winterianus*) containing 1.5% of each essential oil showed higher repellency (91%) against *D. reticulatus* adults than the single essential oils applied at the concentration of 3%.

As shown in the present study, the repellent effect of the emulsions may have depended not only on their qualitative composition but also on the matrix type. The thyme + peppermint oils (from preparation CMC 3) repelled *D. reticulatus* ticks more potently than the same essential oils on the CHIT (chitosan lactate) matrix (emulsion CHIT 3).

The effectiveness of a repellent depends on its physicochemical properties, the dose of the active substance, and the type of formulation. Lavender and geranium oils were reported to exhibit weak repellent activity against *I. ricinus* nymphs when applied in a 1% dilution in 1,2-propanediol, whereas their 30% dilution in 1,2-propanediol had 100% efficacy [60].

## 5. Conclusions

Ten emulsion preparations were developed using natural polymers sourced from renewable materials in combination with essential oils and surfactants—SLP and Tween 80. These preparations exhibited long-term stability and rapid biodegradation in water and were non-toxic to soil microorganisms. With viscosity ranging from 15 to 73 cP, they are well-suited for application in green areas. The wetting angle measurements confirmed their ability to adhere to sprayed surfaces. In tests on two species of ticks, *I. ricinus* and *D. reticulatus*, emulsions containing a blend of lavender + eucalyptus and tea tree essential oils demonstrated the strongest repellent effect. These findings indicate the potential to create an environmentally friendly preparation with essential oils serving as tick repellents, presenting a promising alternative to synthetic products for use in green areas.

## Figures and Tables

**Table 1:** Variants of preparations in the form of emulsion based on sodium carboxymethyl cellulose (CMC) with surfactants and essential oils (**A**); variants of preparations in the form of emulsion based on chitosan (CHIT) with surfactants and essential oils (**B**).

(**A**)				
Polymer base: CMC				
Surfactants: SLP + Tween 80				
Essential oil mixture				
Citronella ^1^ Rosemary ^2^ Geranium ^3^	Lavender ^4^ Eucalyptus ^5^ Tea tree ^6^	Thyme ^7^ Peppermint ^8^	Vanilla ^9^ Lavender	Clove ^10^ Patchouli ^11^
SAMPLE NAME				
CMC 1	CMC 2	CMC 3	CMC 4	CMC 5
(**B**)				
Polymer base: CHIT				
Surfactants: SLP + Tween 80				
Essential oil mixture				
Citronella ^12^ Rosemary ^13^ Geranium ^14^	Lavender ^15^ Eucalyptus ^16^ Tea tree ^17^	Thyme ^18^ Peppermint ^19^	Vanilla ^20^ Lavender	Clove ^21^ Patchouli ^22^
SAMPLE NAME				
CHIT 1	CHIT 2	CHIT 3	CHIT 4	CHIT 5

^1^ Citronella (*Cymbopogon winterianus*), ^2^ Rosemary (*Rosmarinus officinalis*), ^3^ Geranium (*Pelargonium graveolens*), ^4^ Lavender (*Lavandula offcinalis*), ^5^ Ecalyptus (*Eucalyptus globulus*), ^6^ Tea tree (*Melaleuca alternifolia*), ^7^ Thyme (*Thymus vulgaris*), ^8^ Peppermint (*Mentha piperita*), ^9^ Vanilla (*Vanilla Mill.*), ^10^ Clove (*Syzygium aromaticum*), ^11^ Patchouli (*Pogostemon cablin*), ^12^ Citronella (*Cymbopogon winterianus*), ^13^ Rosemary (*Rosmarinus officinalis*), ^14^ Geranium (*Pelargonium graveolens*), ^15^ Lavender (*Lavandula offcinalis*), ^16^ Ecalyptus (*Eucalyptus globulus*), ^17^ Tea tree (*Melaleuca alternifolia*), ^18^ Thyme (*Thymus vulgaris*), ^19^ Peppermint (*Mentha piperita*), ^20^ Vanilla (*Vanilla Mill.*), ^21^ Clove (*Syzygium aromaticum*), ^22^ Patchouli (*Pogostemon cablin*).

**Table 2:** Results of the impact of the composition and degradation of emulsions on the total number of microorganisms in the soil medium.

Day	Reference Sample	CMC 1	CMC 2	CMC 3	CMC 4	CMC 5	CHIT 1	CHIT 2	CHIT 3	CHIT 4	CHIT 5
	Number of Colony Forming Units [cfu]										
0	1.1 × 10^6^	1.1 × 10^6^	1.1 × 10^6^	1.1 × 10^6^	1.1 × 10^6^	1.1 × 10^6^	1.1 × 10^6^	1.1 × 10^6^	1.1 × 10^6^	1.1 × 10^6^	1.1 × 10^6^
1	4.2 × 10^5^	6.5 × 10^5^	1.0 × 10^6^	7.3 × 10^5^	1.1 × 10^6^	6.7 × 10^5^	8.7 × 10^5^	7.3 × 10^5^	6.6 × 10^5^	1.2 × 10^6^	6.5 × 10^5^
4	5.7 × 10^5^	8.7 × 10^5^	7.8 × 10^5^	7.7 × 10^5^	9.2 × 10^5^	8.5 × 10^5^	7.7 × 10^5^	1.0 × 10^6^	8.2 × 10^5^	8.8 × 10^5^	7.1 × 10^5^
7	4.4 × 10^5^	6.6 × 10^5^	5.9 × 10^5^	6.4 × 10^5^	6.1 × 10^5^	5.1 × 10^5^	6.2 × 10^5^	8.7 × 10^5^	7.1 × 10^5^	4.2 × 10^5^	2.6 × 10^5^
8	3.4 × 10^5^	3.8 × 10^5^	4.5 × 10^5^	2.6 × 10^5^	3.3 × 10^5^	3.4 × 10^5^	4.3 × 10^5^	5.2 × 10^5^	5.7 × 10^5^	4.8 × 10^5^	3.8 × 10^5^
11	4.9 × 10^5^	4.2 × 10^5^	6.4 × 10^5^	4.6 × 10^5^	5.7 × 10^5^	6.1 × 10^5^	5.2 × 10^5^	8.2 × 10^5^	8.9 × 10^5^	7.0 × 10^5^	6.2 × 10^5^
14	3.6 × 10^5^	4.8 × 10^5^	5.5 × 10^5^	9.4 × 10^5^	5.7 × 10^5^	5.3 × 10^5^	7.8 × 10^5^	7,5 × 10^5^	7.6 × 10^5^	8.6 × 10^5^	6.5 × 10^5^
15	2.5 × 10^5^	6.7 × 10^5^	4.4 × 10^5^	3.8 × 10^5^	4.2 × 10^5^	4.2 × 10^5^	8.2 × 10^5^	7.9 × 10^5^	7.2 × 10^5^	7.6 × 10^5^	7.4 × 10^5^
18	4.1 × 10^5^	5.8 × 10^5^	5.2 × 10^5^	8.7 × 10^5^	4.9 × 10^5^	5.2 × 10^5^	6.1 × 10^5^	4.0 × 10^5^	4.3 × 10^5^	4.9 × 10^5^	6.6 × 10^5^
21	5.0 × 10^5^	5.1 × 10^5^	5.6 × 10^5^	6.5 × 10^5^	4.4 × 10^5^	4.9 × 10^5^	5.4 × 10^5^	5.6 × 10^5^	5.8 × 10^5^	6.2 × 10^5^	7.2 × 10^5^
22	4.1 × 10^5^	1.6 × 10^5^	5.8 × 10^5^	5.0 × 10^5^	3.9 × 10^5^	6.9 × 10^5^	7.3 × 10^5^	6.3 × 10^5^	2.0 × 10^5^	5.7 × 10^5^	5.3 × 10^5^
28	3.8 × 10^5^	3.0 × 10^5^	5.7 × 10^5^	2.7 × 10^5^	6.8 × 10^5^	5.5 × 10^5^	4.3 × 10^5^	6.8 × 10^5^	6.3 × 10^5^	7.0 × 10^5^	3.7 × 10^5^

**Table 3:** Results of the assessment of the stability of emulsion preparations—centrifuge method.

Symbol of Sample	Centrifugal Stability 4000 rpm Organoleptic Evaluation		
	10 min	20 min	30 min
CMC 1	Stable sample	Stable sample	Stable sample
CMC 2	Stable sample	Stable sample	Stable sample
CMC 3	Stable sample	Stable sample	Stable sample
CMC 4	Stable sample	Stable sample	Stable sample
CMC 5	Stable sample	Stable sample	Stable sample
CHIT 1	Stable sample	Stable sample	Stable sample
CHIT 2	Stable sample	Stable sample	Stable sample
CHIT 3	Stable sample	Stable sample	Stable sample
CHIT 4	Stable sample	Stable sample	Stable sample
CHIT 5	Stable sample	Stable sample	Stable sample

**Table 4:** Results of the assessment of the stability of emulsion preparations—thermal method.

Symbol of Sample	0 Days	T = 25 ± 1 °C, RH—29 ± 1%				
		Organoleptic Evaluation After Time				
		7 Days	14 Days	21 Days	30 Days	60 Days
CMC 1	Homogeneous emulsion	Stable sample, No change	Stable sample, No change	Stable sample, No change	Stable sample No change	Stable sample No change
CMC 2	Homogeneous emulsion	Stable sample, No change	Stable sample No change	Stable sample, No change	Stable sample No change	Stable sample No change
CMC 3	Homogeneous emulsion	Stable sample, No change	Stable sample No change	Stable sample, No change	Stable sample No change	Stable sample No change
CMC 4	Homogeneous emulsion	Stable sample, No change	Stable sample No change	Stable sample, No change	Stable sample No change	The sample succumbed to sedimentation (a reversible phenomenon)
CMC 5	Homogeneous emulsion	Stable sample, No change	Stable sample No change	Stable sample, No change	Stable sample No change	The sample succumbed to sedimentation (a reversible phenomenon)
CHIT 1	Homogeneous emulsion	Stable sample, No change	Stable sample No change	Stable sample, No change	Stable sample No change	The sample succumbed to sedimentation (a reversible phenomenon)
CHIT 2	Homogeneous emulsion	Stable sample, No change	Stable sample No change	Stable sample, No change	Stable sample No change	The sample succumbed to sedimentation (a reversible phenomenon)
CHIT 3	Homogeneous emulsion	Stable sample, No change	Stable sample No change	Stable sample, No change	Stable sample No change	Stable sample No change
CHIT 4	Homogeneous emulsion	Stable sample, No change	Stable sample No change	Stable sample, No change	Stable sample No change	The sample succumbed to sedimentation (a reversible phenomenon
CHIT 5	Homogeneous emulsion	Stable sample, No change	Stable sample No change	Stable sample, No change	Stable sample No change	The sample succumbed to sedimentation (a reversible phenomenon)

T—temperature, RH—relative humidity.

## Data Availability

The data presented in this study are available on request from the corresponding authors.

## Supplementary Materials

The following supporting information can be downloaded at: https://www.mdpi.com/article/10.3390/insects15120931/s1, Supplementary Material File S1: The GC-MS chromatographic analysis of the studied essential oils, including the mass spectra of the main compounds; Table S1: Behavior of *Dermacentor reticulatus* adults under the influence of mixture CMC 1 (citronella + rosemary + geranium); Table S2: Behavior of *Dermacentor reticulatus* adults under the influence of mixture CMC 2 (lavender + eucalyptus + tea tree); Table S3: Behavior of *Dermacentor reticulatus* adults under the influence of mixture CMC 3 (thyme + peppermint); Table S4: Behavior of *Dermacentor reticulatus* adults under the influence of mixture CMC 4 (vanilla + lavender); Table S5: Behavior of *Dermacentor reticulatus* adults under the influence of mixture CMC 5 (clove + patchouli); Table S6: Behavior of *Dermacentor reticulatus* adults under the influence of mixture CHIT 1 (citronella + rosemary + geranium); Table S7: Behavior of *Dermacentor reticulatus* adults under the influence of mixture CHIT 2 (lavender + eucalyptus + tea tree); Table S8: Behavior of *Dermacentor reticulatus* adults under the influence of mixture CHIT 3 (thyme + peppermint); Table S9: Behavior of *Dermacentor reticulatus* adults under the influence of mixture CHIT 4 (vanilla + lavender); Table S10: Behavior of *Dermacentor reticulatus* adults under the influence of mixture CHIT (clove + patchouli); Table S11: Behavior of *Ixodes ricinus* females under the influence of selected emulsions; Table S12: Behavior of *Dermacentor reticulatus* adults under the influence of sodium carboxymethyl cellulose (CMC); Table S13: Behavior of *Dermacentor reticulatus* adults under the influence of chitosan lactate (CHIT) [97].
