# Supplementary material for: Ecological Repellent Preparations Based on Natural Polymers with the Addition of Essential Oils Acting on Ticks
Source: Insects. 2024 Nov 27;15(12):931. doi: 10.3390/insects15120931 (PMC11678291; doi:10.3390/insects15120931)
Supplement: Supplementary file 1 [file insects-15-00931-s001.zip › insects-3190285-supplementary.pdf]

insects

MDPI

Supplementary Material 1:

The GC-MS chromatographic analysis of the studied essential oils, including the mass spectra of the main compounds

Methodology

Gas Chromatography-Mass Spectrometry (GC-MS) analysis of essential oils was performed using an Agilent Technologies 7890A GC system coupled with a 5975C mass selective detector. The chromatographic separation was achieved with a ZB-SemiVolatiles column (Phenomenex, USA), featuring the following dimensions: 30 m in length, 0.25 mm inner diameter, and 0.25 μm film thickness. Dilutions of essential oil solutions were prepared at a ratio of 1:1000 (1 μL/mL), using methanol as the solvent.

GC-MS Parameters:

- Injector Temperature: 260 °C
- Injection Mode: Splitless
- Oven Temperature Program:
  - Initial temperature: 80 °C, held for 2 minutes
  - Ramp: 10 °C/min
  - Final temperature: 300 °C
- Ion Source Temperature: 230 °C
- Quadrupole Temperature: 150 °C
- Carrier Gas Flow Rate: 1 mL/min (helium)
- Data Acquisition Mode: SCAN
- Agilent Spectral Library: NIST MSSearch 2.0.

1. *Results*

Compounds obtained from the GC–MS analysis of Essential oils.

	Compound Name/Structure	CAS number	Formula	Molecular Weight	Retention Time (min)	Key Compounds Detected in GC-MS Mass Spectra with m/z Ratio [1]
1	**Citronella (*Cymbopoogon vinterianus*)**					
	Citronellal 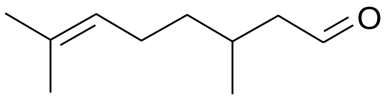	106-23-0	$C_{10}H_{18}O$	154	5.01	69, 41, 95
	D-Limonene	5989-27-5	$C_{10}H_{16}$	136	3.73	68, 93, 67

	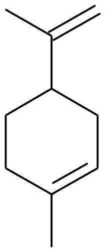					
	Geraniol	106-24-1	$C_{10}H_{18}O$	154	6.21	69, 41, 68
	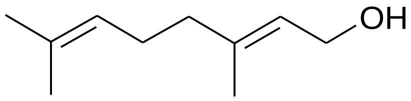					
2	**Rosemary (*Rosmarini officinalis*)**					
	Eucalyptol	470-82-6	$C_{10}H_{18}O$	154	3.79	43, 81, 93
	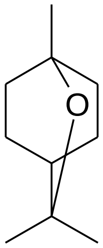					
	Camphor	464-49-3	$C_{10}H_{16}O$	MW = 152	RT 5.08	95, 81, 108
	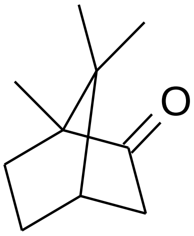					
	Alfa-Pinen	7785-70-8	$C_{10}H_{16}$	136	3.55	93, 91, 92
	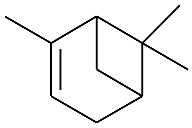					
	Borneol	464-45-9	$C_{10}H_{18}O$	154	5.28	95, 110, 41
	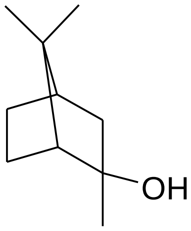					
3	**Geranium (*Pelargonium graveolens*)**					

	Citronellol	106-22-9	$C_{10}H_{20}O$	156	5.91	69, 41, 67
	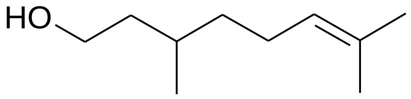					
	Geraniol	106-24-1	$C_{10}H_{18}O$	154	6.21	69, 41, 68
	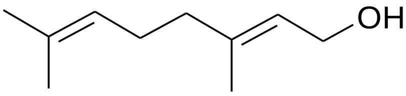					
4	**Lavender (*Lavendula offcinalis*)**					
	Linalol	78-70-6	$C_{10}H_{18}O$	154	4.41	71, 93, 55
	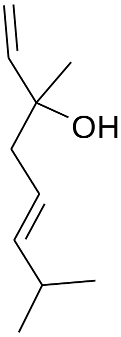					
	Caryophynelle	87-44-5	$C_{15}H_{24}$	204	8.52	93, 133, 91
	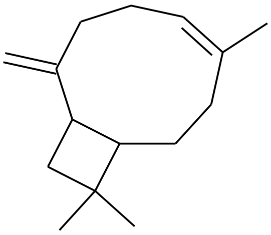					
5	**Ecalyptus (*Eucalipti globulus*)**					
	Eukalyptol	470-82-6	$C_{10}H_{18}O$	154	3.79	43, 81, 93
	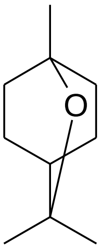					
	Alfa-Pinene	7785-70-8	$C_{10}H_{16}$	136	3.55	93, 91, 92
	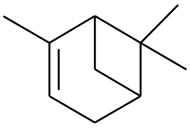					

6	**Tea tree (*Malaleuca altemifolia*)**					
	Terpinen-4-ol 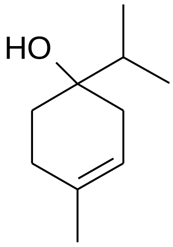	562-74-3	$C_{10}H_{18}O$	154	5.44	71, 111, 93
	gamma-Terpinen 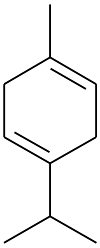	99-85-4	$C_{10}H_{16}$	136	4.01	93, 91, 77
7	**Thyme (*Thymus vulgaris*)**					
	Thymol 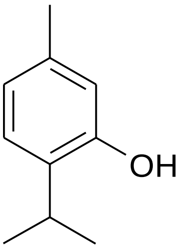	89-83-8	$C_{10}H_{14}O$	150	6.72	135, 150, 91
	Karvakrol 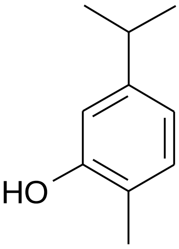	499-75-2	$C_{10}H_{14}O$	150	6.84	135, 150, 91
8	**Peppermint (*Mentha piperita*)**					
	Menthol 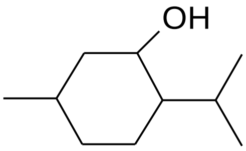	15356-70-4	$C_{10}H_{20}O$	156	5.39	71, 81, 95
	Menthone	10458-14-7	$C_{10}H_{18}O$	154	5.13	112, 69, 41

	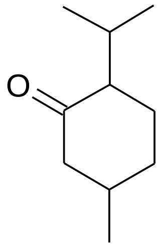					
9	**Vanilla (*Vanilla Mill.*)**					
	Vanillin 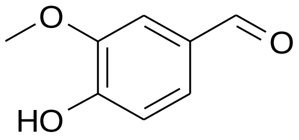	15356-70-4	$C_{10}H_{20}O$	156	5.39	71, 81, 95
	Ethyl Vanillin 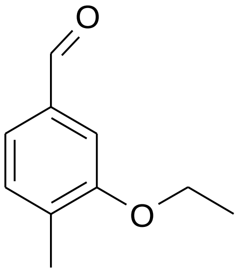	10458-14-7	$C_{10}H_{18}O$	154	5.13	112, 69, 41
10	**Cloves (*Eugenia caryophylus*)**					
	Eugenol 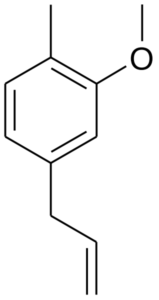	97-53-0	$C_{10}H_{12}O_2$	164	7.59	164, 103, 77
	Aceto Eugenol	93-28-7	$C_{12}H_{14}O_3$	206	9.53	164, 149, 43

	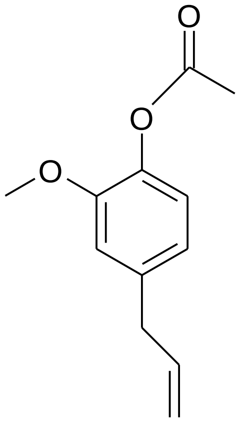					
11	**Patchouli (*Pogostemon cablin*)**					
	Patchoulol (patchouli alcohol)	5986-55-0	$C_{15}H_{26}O$	222	11.59	41, 43, 83
	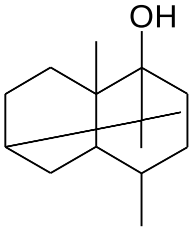					
	alfa-Bulnesene	3691-11-0	$C_{15}H_{24}$	204	9.49	107, 108, 93
	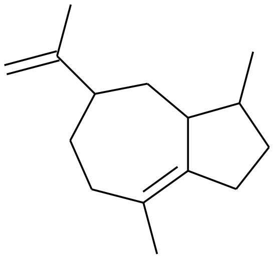					

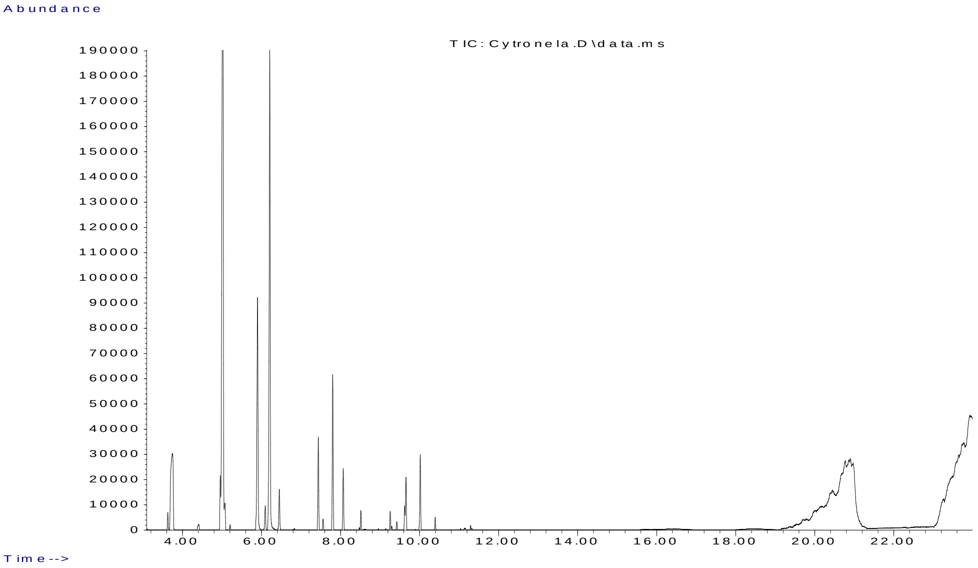

Figure 1. Chromatogram of the gas chromatography–mass spectroscopy analysis of the Citronella (*Cymbopoogon vinterianus*).

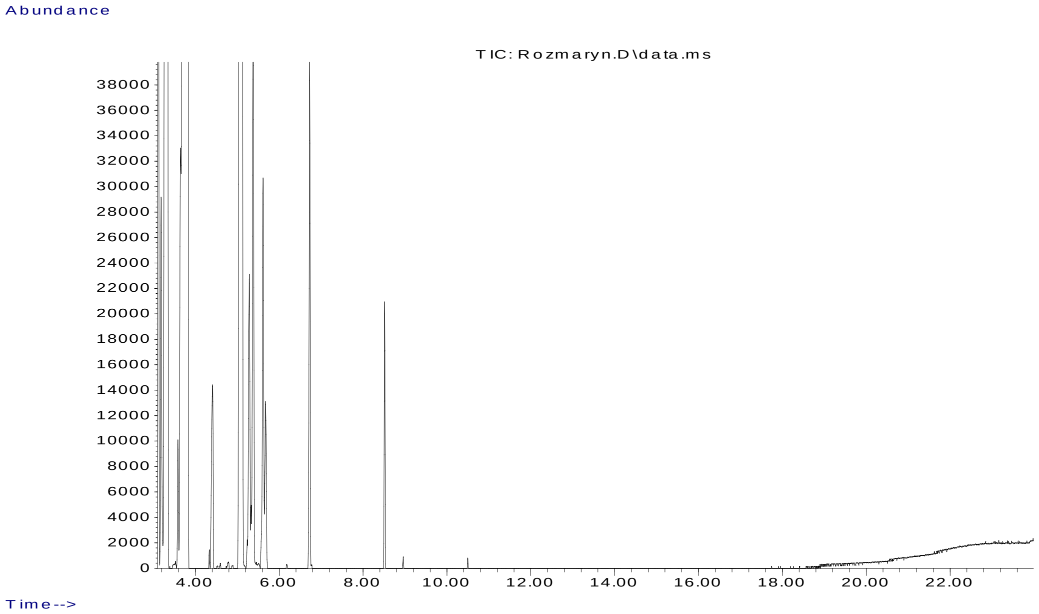

Figure 2. Chromatogram of the gas chromatography–mass spectroscopy analysis of the Rosemary (*Rosmarini officinalis*).

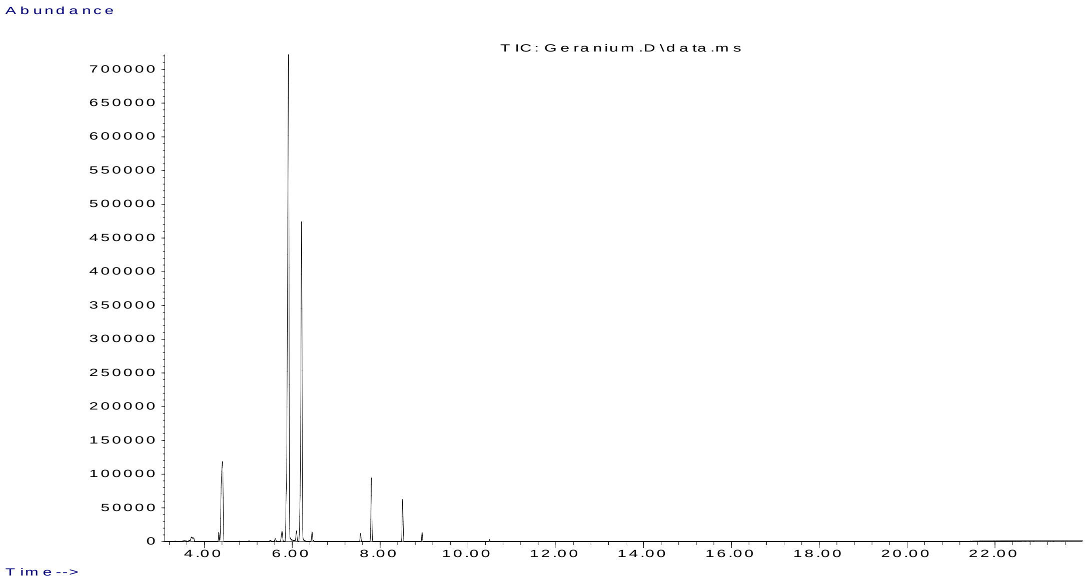

Figure 3. Chromatogram of the gas chromatography–mass spectroscopy analysis of the Geranium (*Pelargonium graveolens*).

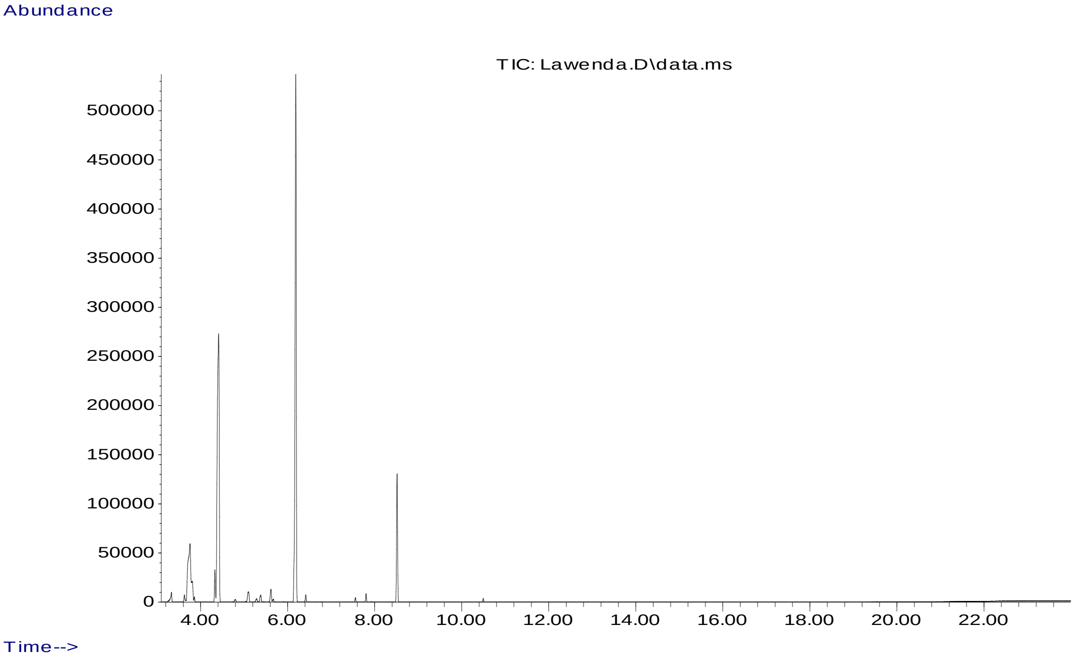

Figure 4. Chromatogram of the gas chromatography–mass spectroscopy analysis of the Lavender (*Lavendula offcinalis*).

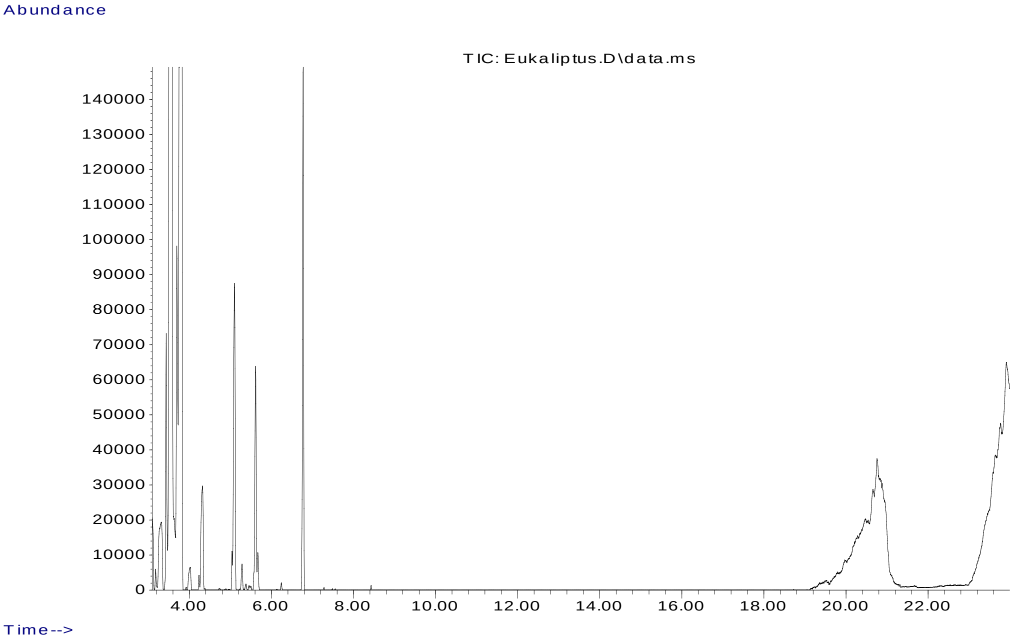

Figure 5. Chromatogram of the gas chromatography–mass spectroscopy analysis of the Ecalyptus (*Eucalipti globulus*).

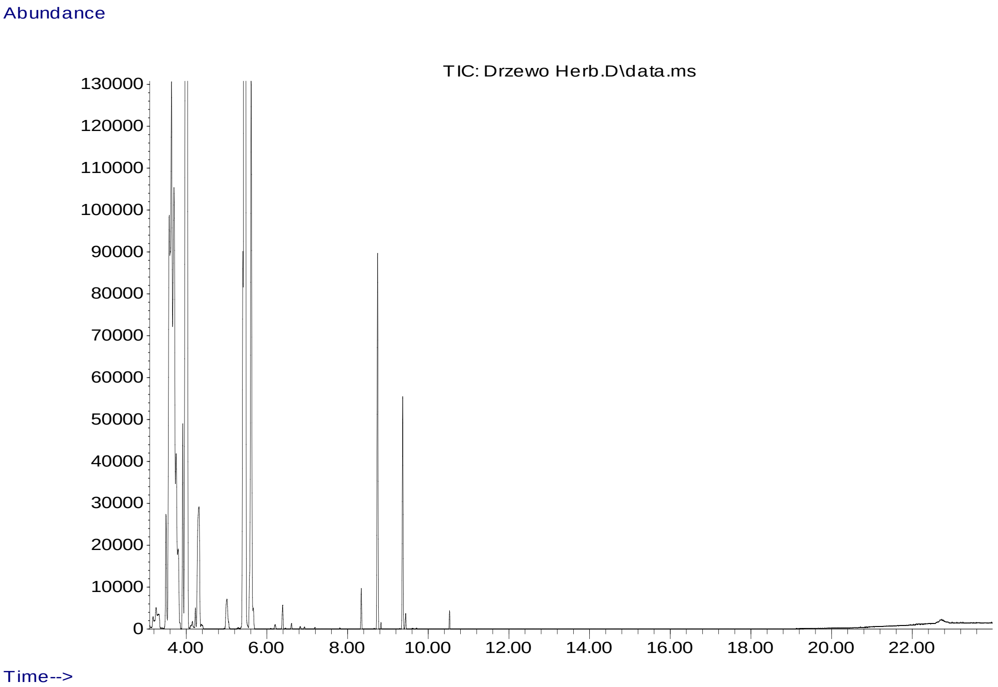

Figure 6. Chromatogram of the gas chromatography–mass spectroscopy analysis of the Tea tree (*Malaleuca altemifolia*).

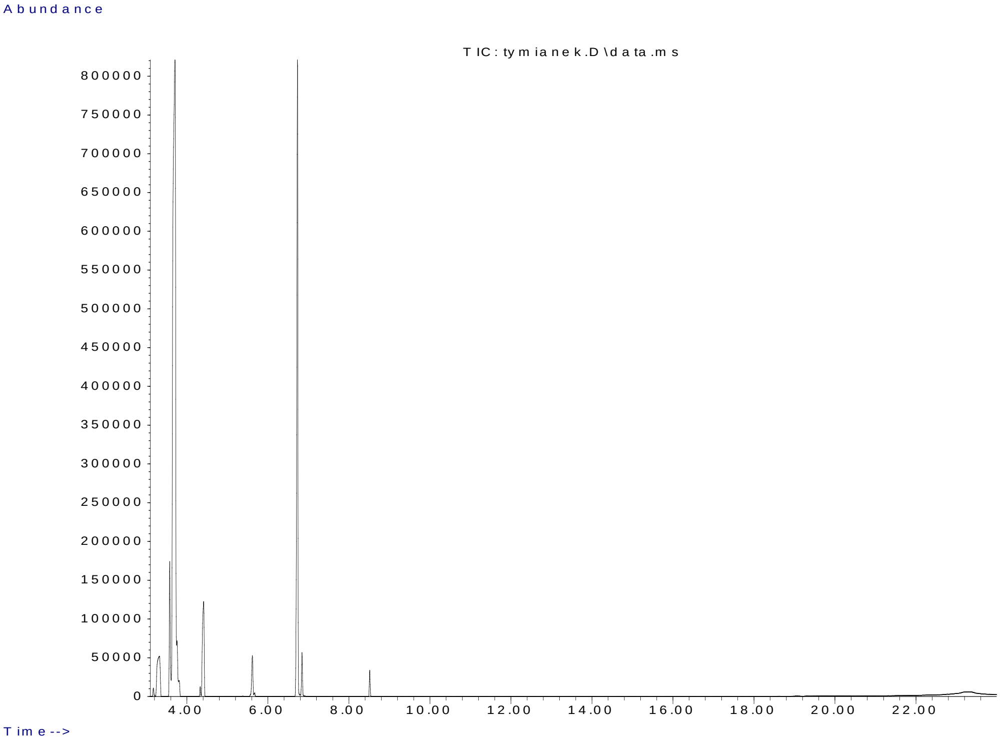

Figure 7. Chromatogram of the gas chromatography–mass spectroscopy analysis of the Thyme (*Thymus vulgaris*).

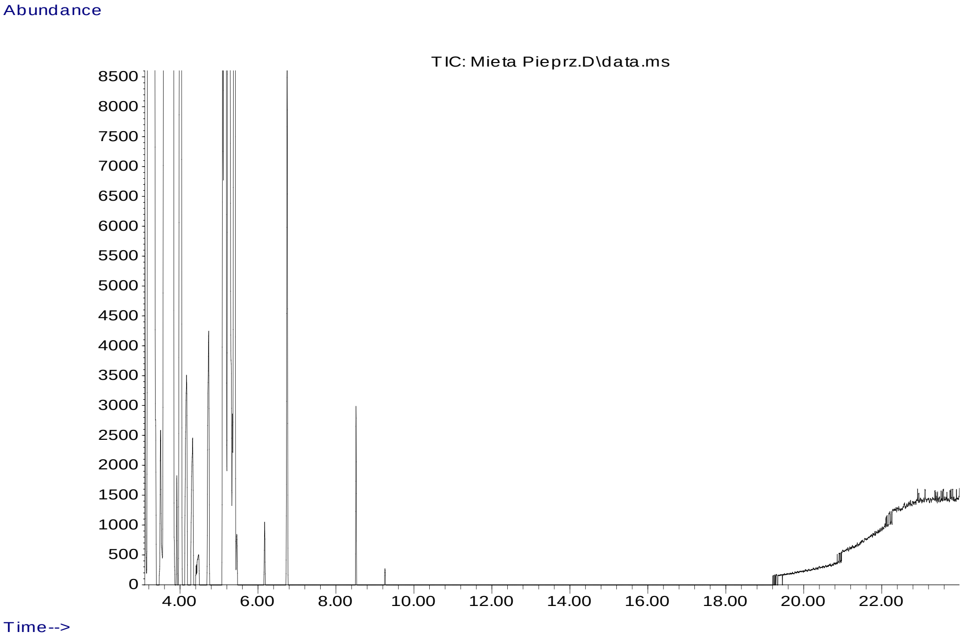

Figure 8. Chromatogram of the gas chromatography–mass spectroscopy analysis of the Peppermint (*Mentha piperita*).

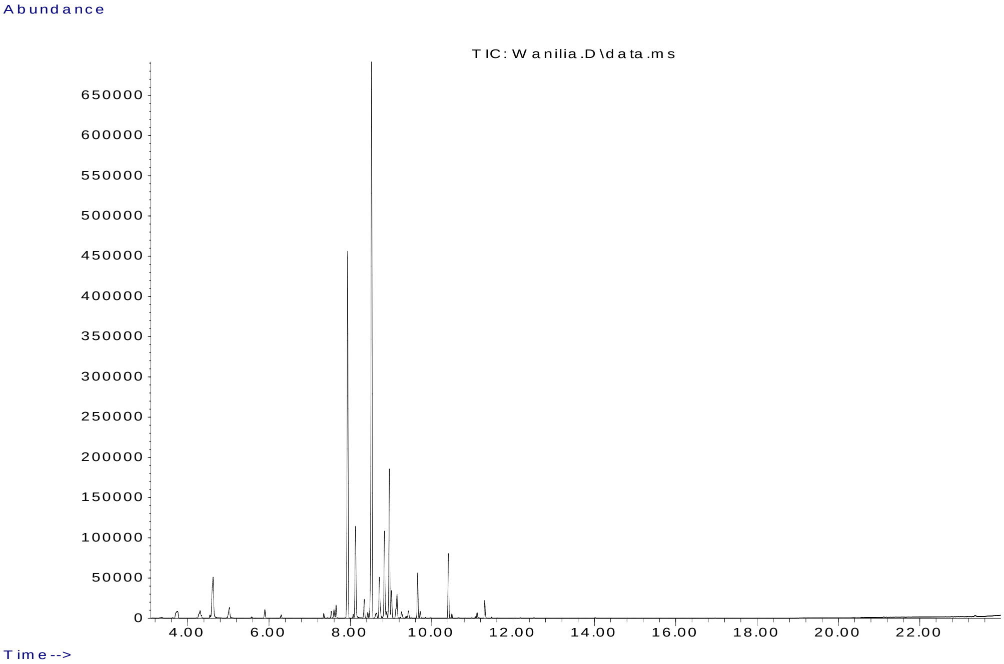

Figure 9. Chromatogram of the gas chromatography–mass spectroscopy analysis of the Vanilla (*Vanilla Mill*).

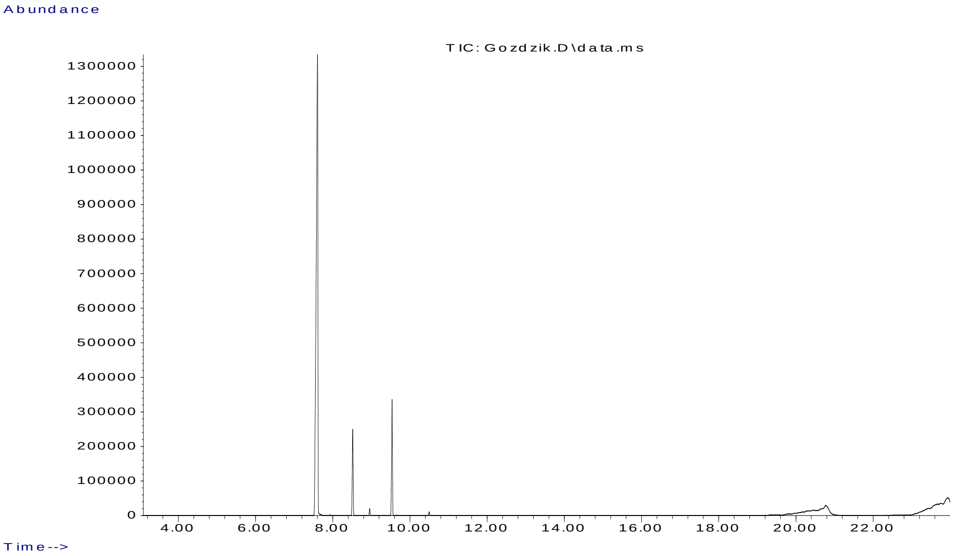

Figure 10. Chromatogram of the gas chromatography–mass spectroscopy analysis of the Cloves (*Eugenia caryophylus*).

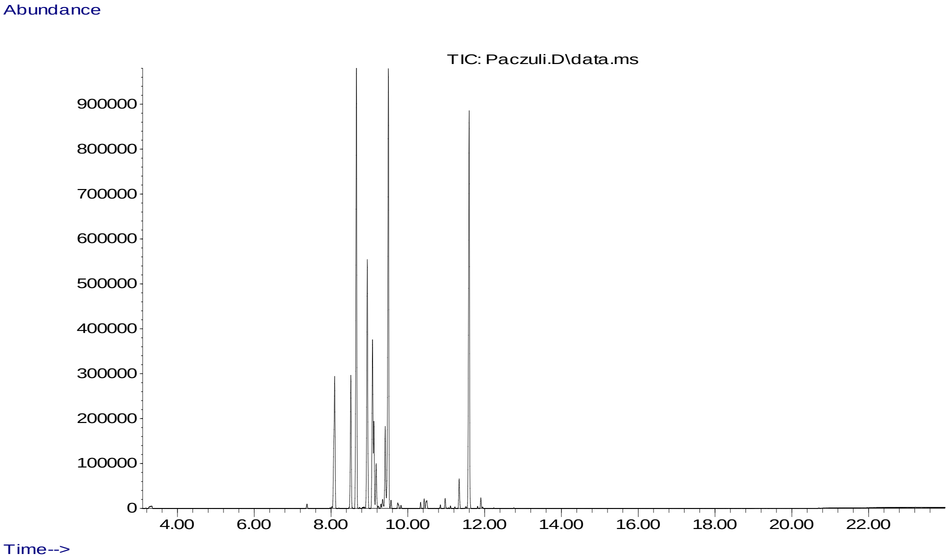

Figure 11. Chromatogram of the gas chromatography–mass spectroscopy analysis of the Patchouli (P*ogostemon cablin*).

Compound Name	Mass spectrum of obtained compound	Mass Spectrum of the Target Compound – Agilent Spectral Library
Citronellal	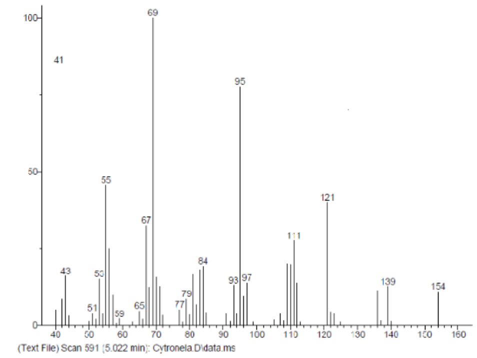	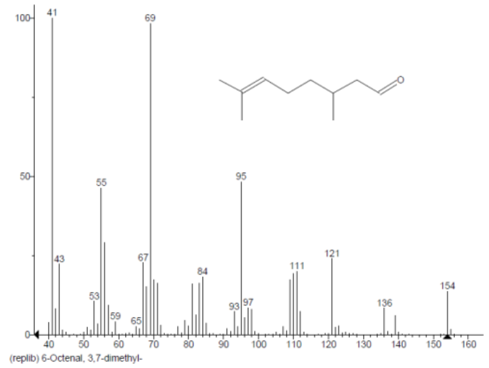
D-Limonene	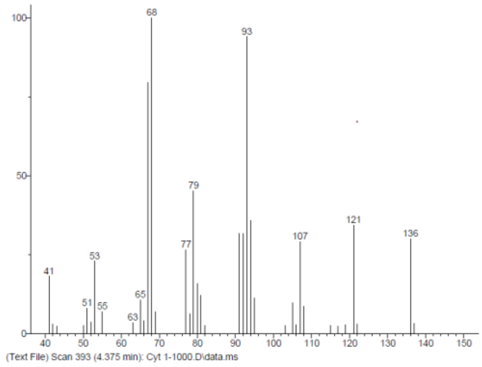	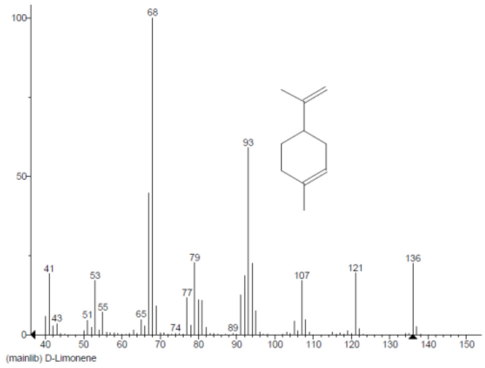
Geraniol	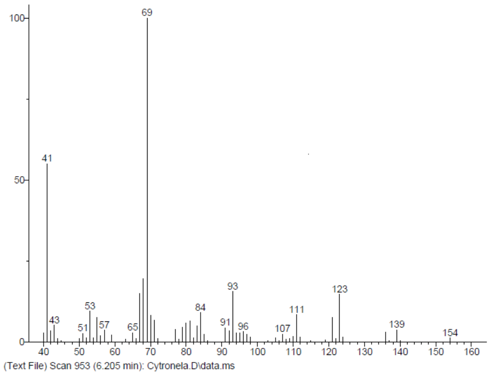	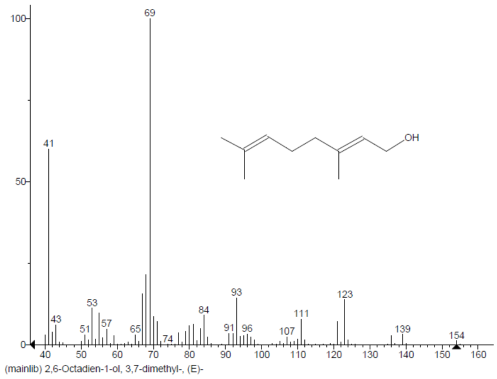

Figure 12. Mass spectra of the Citronella (*Cymbopoogon vinterianus*) - Key Compounds.

Compound Name	Mass spectrum of obtained compound	Mass Spectrum of the Target Compound – Agilent Spectral Library
Eucalyptol	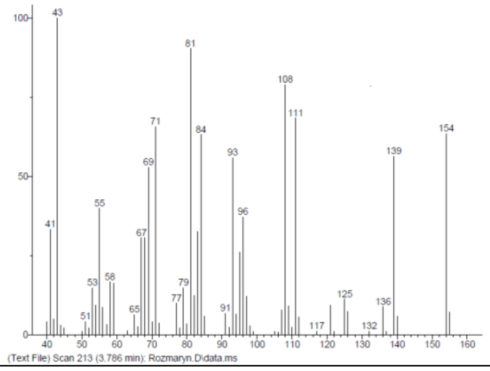	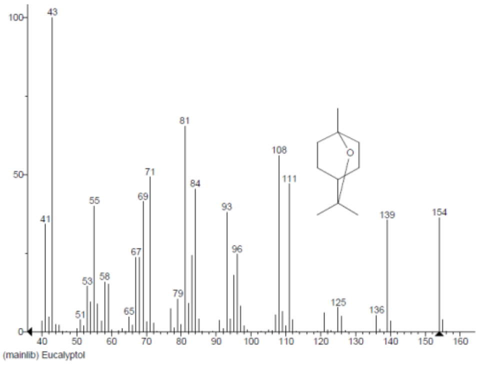
Camphor	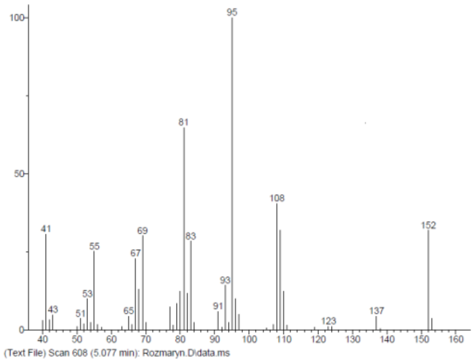	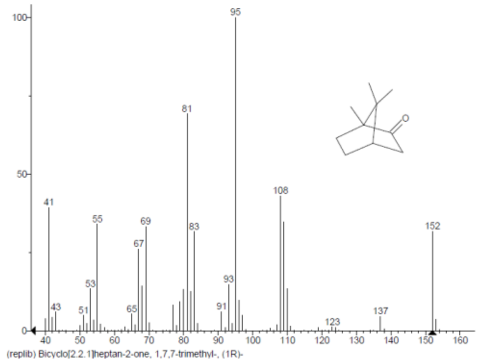
Alfa-Pinen	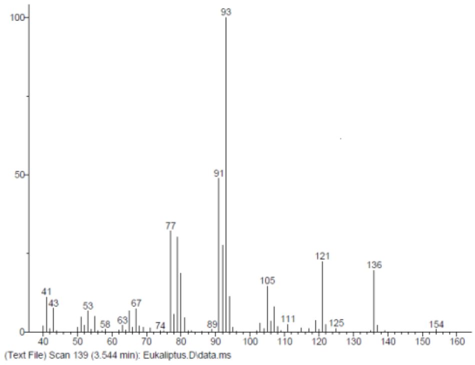	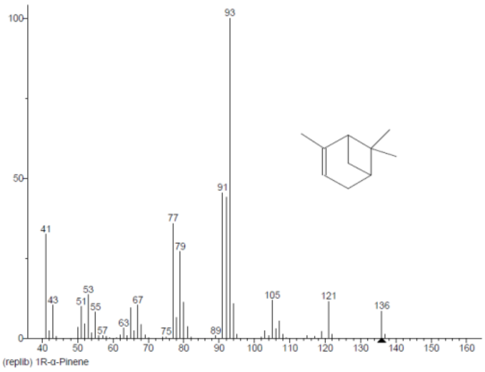
Borneol	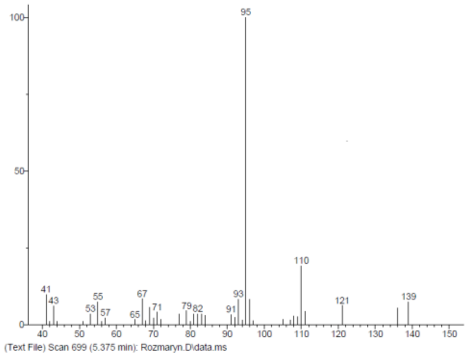	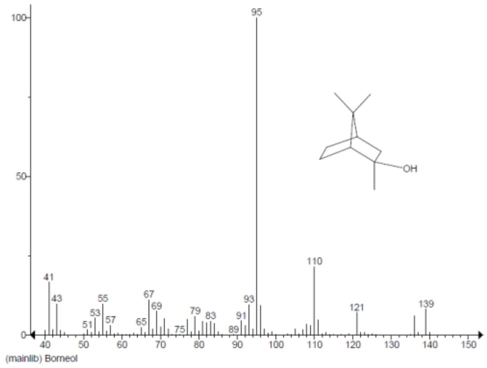

Figure 13. Mass spectra of the Rosemary (*Rosmarini officinalis*) - Key Compounds.

Compound Name	Mass spectrum of obtained compound	Mass Spectrum of the Target Compound – Agilent Spectral Library
Citronellol	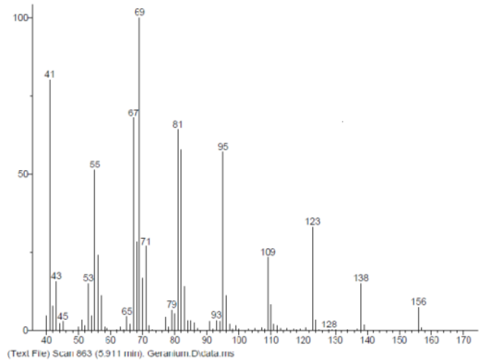	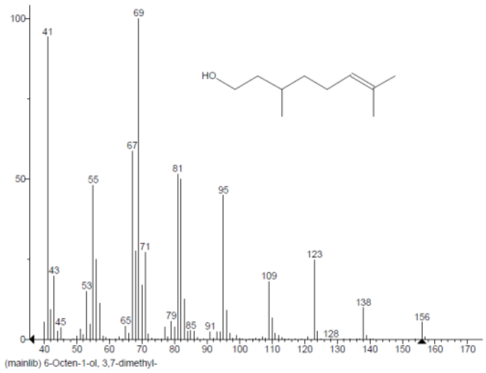
Geraniol	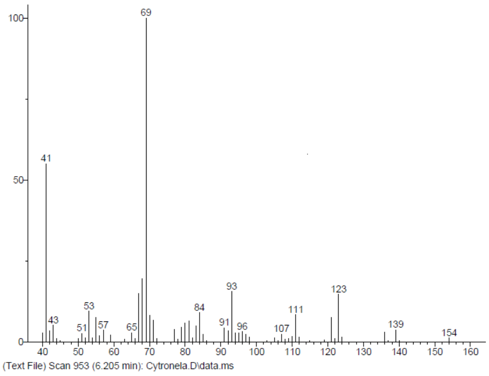	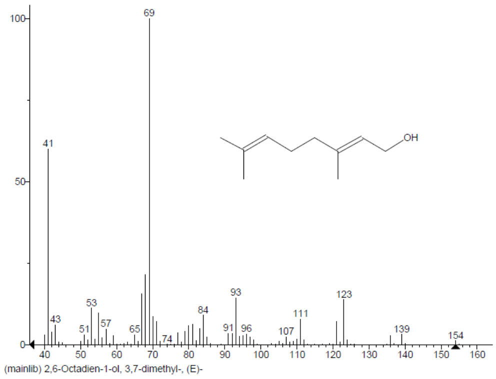

Figure 14. Mass spectra of the Geranium (*Pelargonium graveolens*) - Key Compounds.

Compound Name	Mass spectrum of obtained compound	Mass Spectrum of the Target Compound – Agilent Spectral Library
Linalol	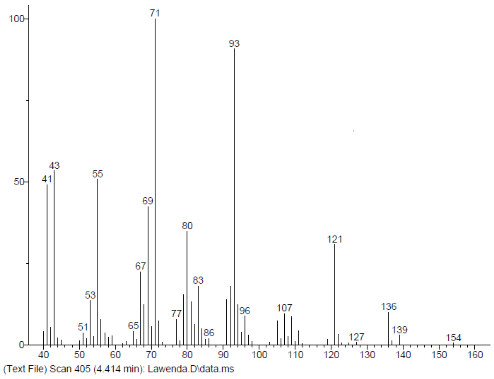	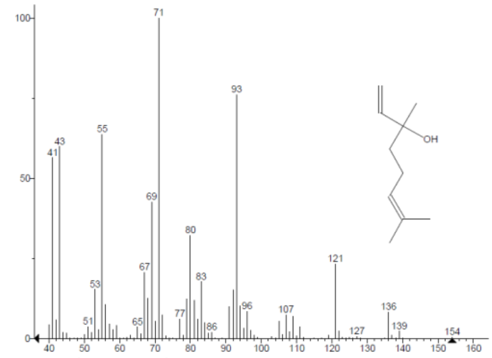
Caryophynelle	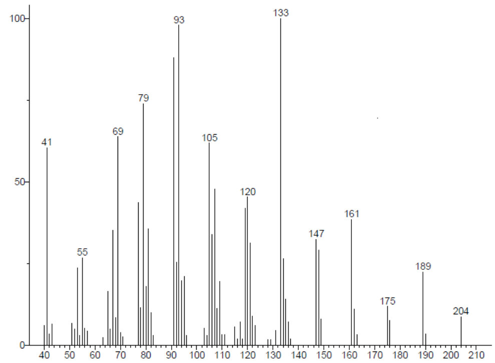	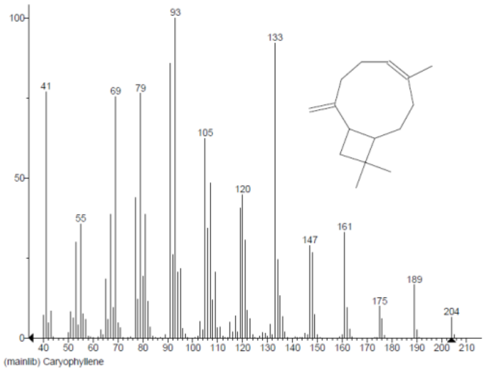

Figure 15. Mass spectra of the Lavender (*Lavendula offcinalis*) - Key Compounds.

Compound Name	Mass spectrum of obtained compound	Mass Spectrum of the Target Compound – Agilent Spectral Library
Eucalyptol	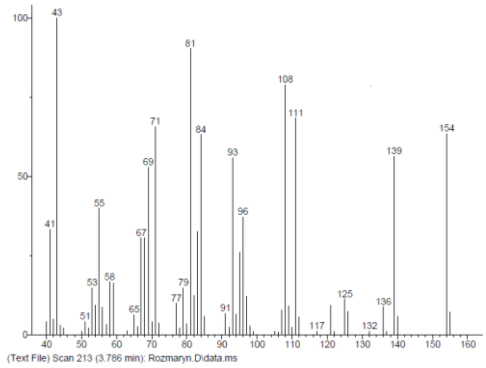	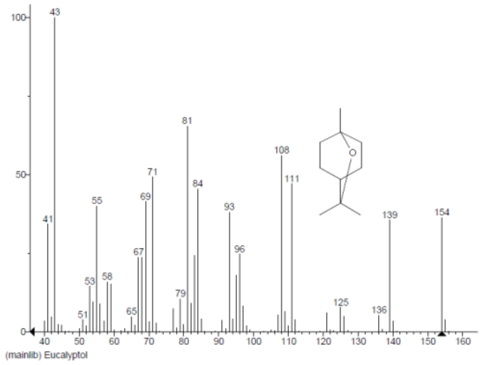
Alfa-Pinen	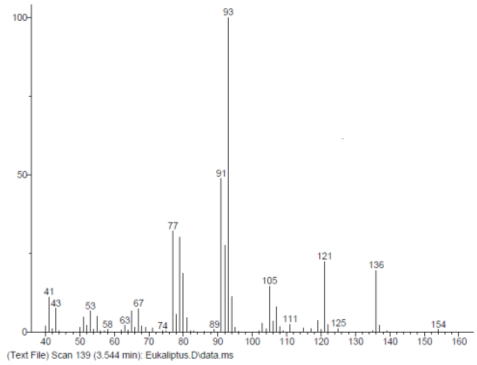	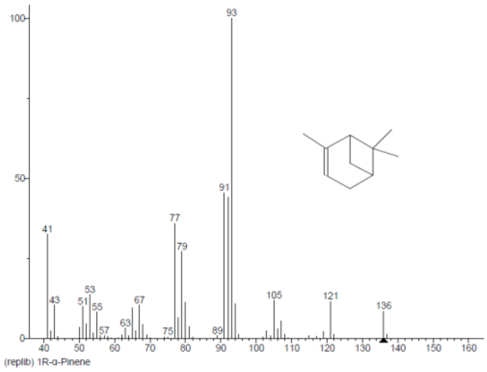

Figure 16. Mass spectra of the Ecalyptus (*Eucalipti globulus*) - Key Compounds.

Compound Name	Mass spectrum of obtained compound	Mass Spectrum of the Target Compound – Agilent Spectral Library
Terpinen-4-ol	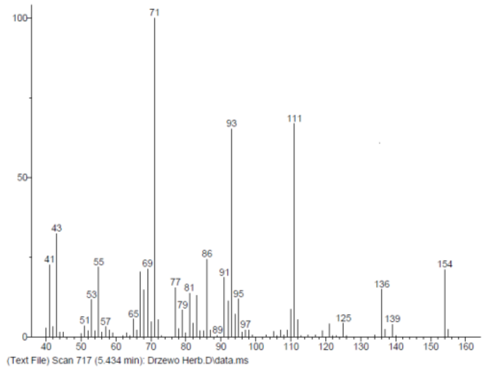	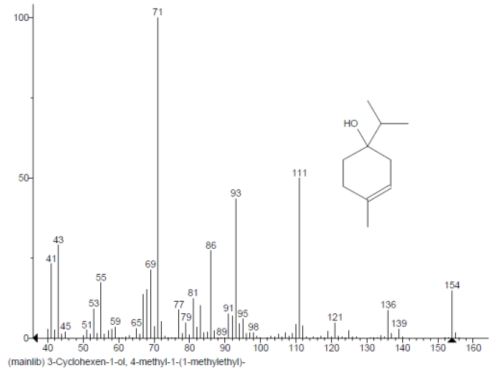
gamma-Terpinen	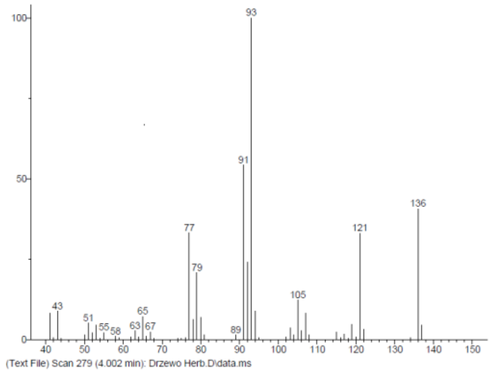	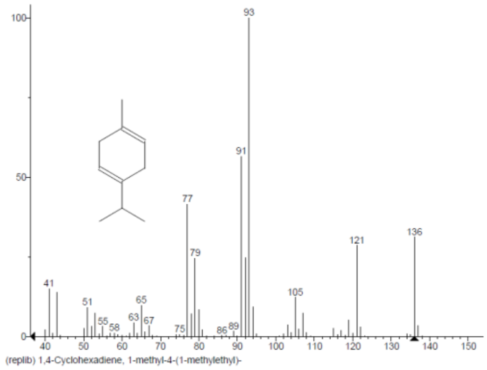

Figure 17. Mass spectra of the Tea tree (*Malaleuca altemifolia*) - Key Compounds.

Compound Name	Mass spectrum of obtained compound	Mass Spectrum of the Target Compound – Agilent Spectral Library
Thymol	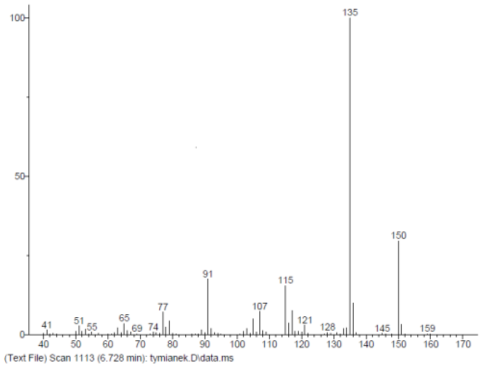	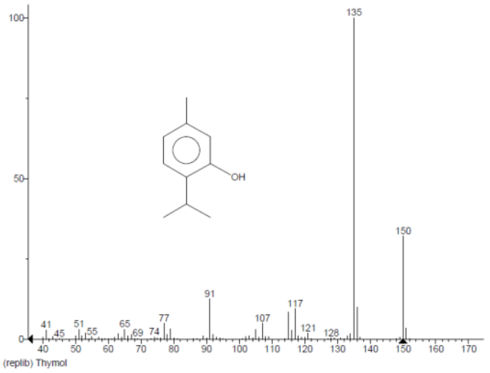
Karvakrol	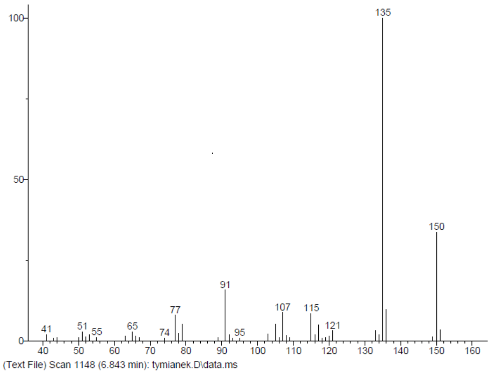	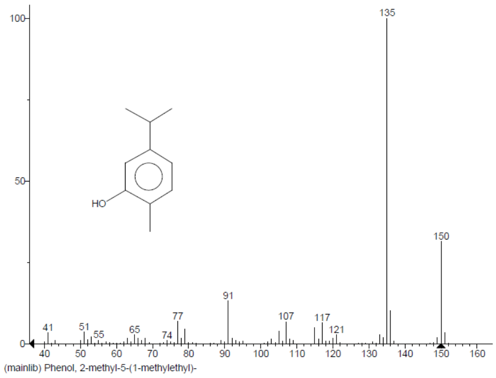

Figure 18. Mass spectra of the Thyme (*Thymus vulgaris*) - Key Compounds.

Compound Name	Mass spectrum of obtained compound	Mass Spectrum of the Target Compound – Agilent Spectral Library
Menthol	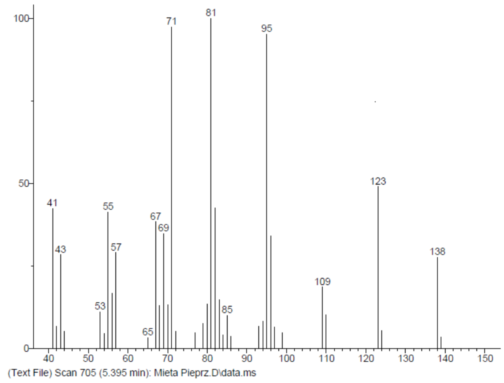	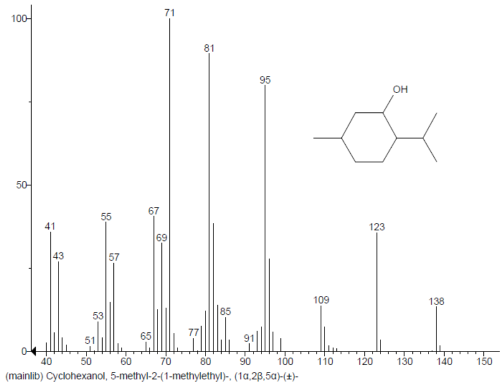
Menthone	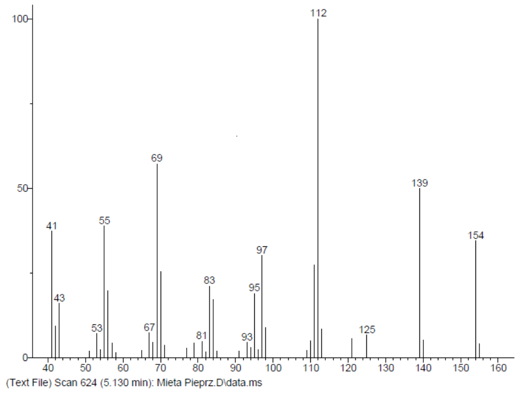	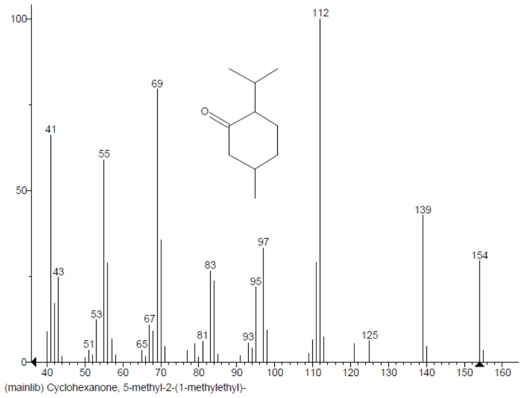

Figure 19. Mass spectra of the Peppermint (*Mentha piperita*) - Key Compounds.

Compound Name	Mass spectrum of obtained compound	Mass Spectrum of the Target Compound – Agilent Spectral Library
Vanillin	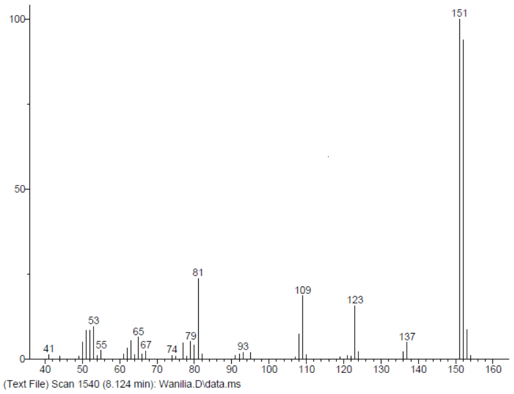	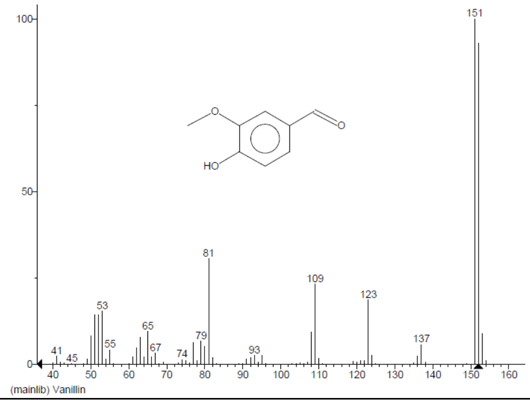
Ethyl Vanillin	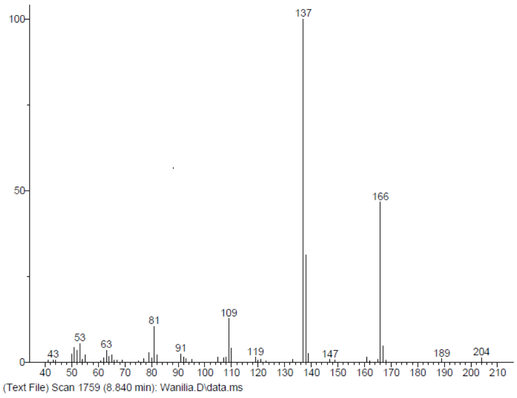	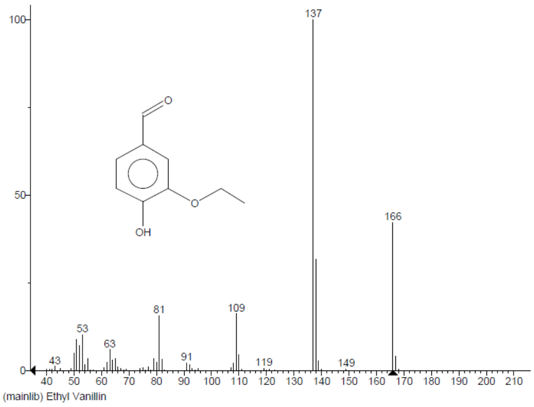

Figure 20. Mass spectra of the Vanilla (*Vanilla Mill.*) - Key Compounds.

Compound Name	Mass spectrum of obtained compound	Mass Spectrum of the Target Compound – Agilent Spectral Library
Eugenol	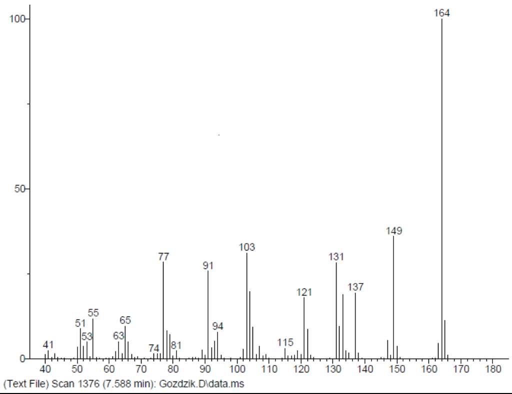	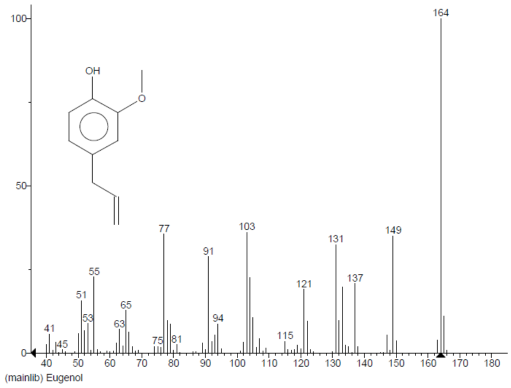
Aceto Eugenol	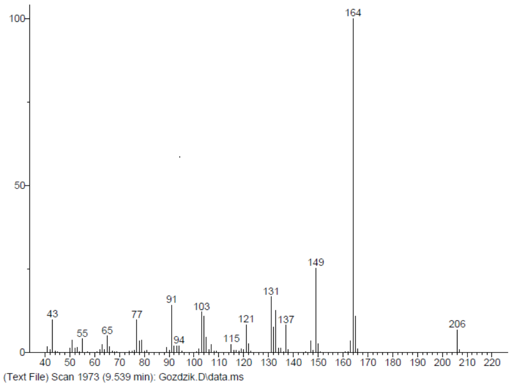	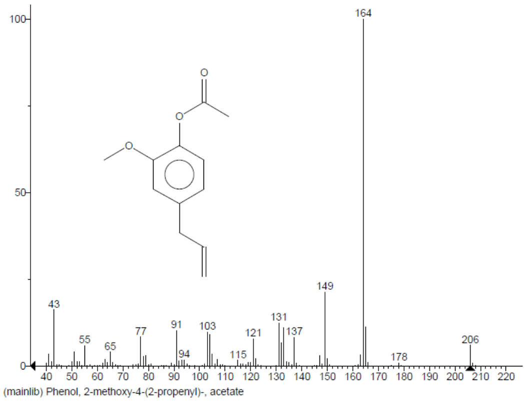

Figure 21. Mass spectra of the Cloves (*Eugenia caryophylus*) - Key Compounds.

Compound Name	Mass spectrum of obtained compound	Mass Spectrum of the Target Compound – Agilent Spectral Library
Patchoulol (patchouli alcohol)	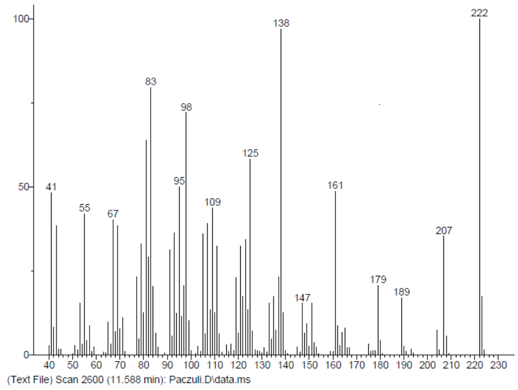	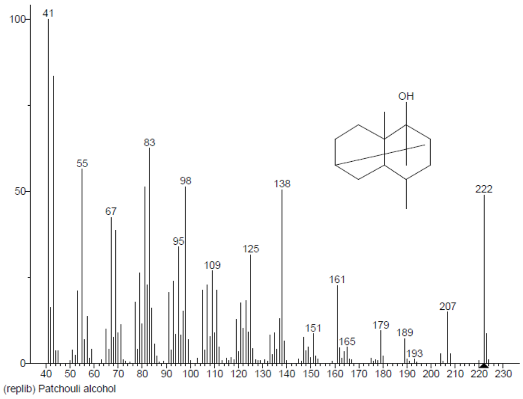
alfa-Bulnesene	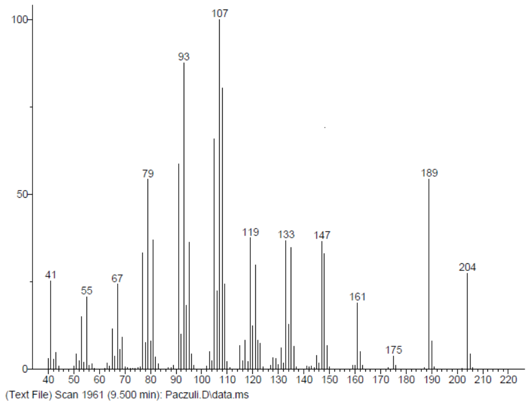	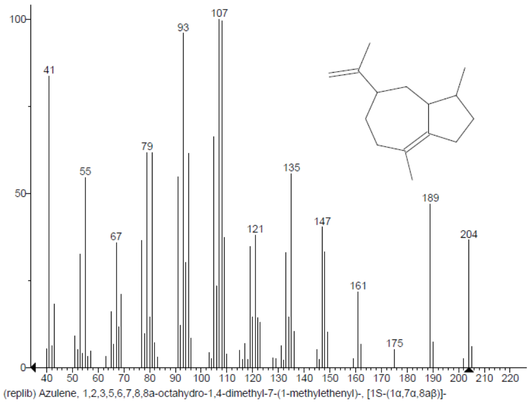

Figure 22. Mass spectra of the Patchouli (*Pogostemon cablin*) - Key Compounds.

References:

1. Adams, R. P. Identification of essential oil components by gas chromatography/ mass spectrometry, 4th Edition. Baylor University, Allured Publ., Carol Stream, 2007.

Table S1. Behavior of *Dermacentor reticulatus* adults under the influence of mixture CMC 1 (citronella + rosemary + geranium). Number (%) of specimens present at a distance greater than/equal to 3 cm from the source of the tested emulsion.

T (min)	CMC 1						Controls					
	500 µl/ 7 cm²			250 µl/ 7 cm²			500 µl/ 7 cm²			250 µl/ 7 cm²		
	Females N (%)	Males N (%)	Adults* N (%)	Females N (%)	Males N (%)	Adults N (%)	Females N (%)	Males N (%)	Adults N (%)	Females N (%)	Males N (%)	Adults N (%)
**15**	8 (100)	4 (100)	12 (100)	8 (100)	4 (100)	12 (100)	2 (25)	1 (25)	3 (25)	2 (25)	0	2 (16.6)
**30**	8 (100)	4 (100)	12 (100)	7 (87.5)	4 (100)	11 (91.6)	1 (12.5)	0	1 (8.3)	1 (12.5)	0	1 (8.3)
**45**	8 (100)	3 (75) **	11 (91.6)	7 (87.5)	4 (100)	11 (91.6)	2 (25)	0	2 (16.6)	0	0	0
**60**	8 (100)	3 (75)	11 (91.6)	5 (62.5)	3 (75)	8 (66.6)	1 (12.5)	0	1 (8.3)	1 (12.5)	0	1 (8.3)
**90**	7 (87.5)	2 (50)	9 (75.0)	4 (50)	3 (75)	7 (58.3)	2 (25)	0	2 (16.6)	1 (12.5)	0	1 (8.3)
**120**	7 (87.5)	3 (75)	10 (83.3)	3 (37.5)	3 (75)	6 (50)	2 (25)	0	2 (16.6)	0	0	0
**150**	7 (87.5)	3 (75)	10 (83.3)	3 (37.5)	3 (75)	6 (50)	3 (37.5)	0	3 (25)	1 (12.5)	0	1 (8.3)
**180**	8 (100)	4 (100)	12 (100)	3 (37.5)	3 (75)	6 (50)	2 (25)	0	2 (16.6)	1 (12.5)	1 (25)	2 (16.6)
**240**	7 (87.5)	4 (100)	11 (91.6)	3 (37.5)	2 (50)	5 (41.6)	3 (37.5)	0	3 (25)	1 (12.5)	1 (25)	2 (16.6)

T – Time after application of the tested emulsion/H_2O, N- number of ticks, * in every experimental group, as in the single control, 12 adults of *D. reticulatus* (8 females and 4 males) were used, ** all other specimens not included in the table were located at the site of application of the tested emulsion/H_2O and/or at a distance less than 3 cm from the site of its application.

Table S2. Behavior of *Dermacentor reticulatus* adults under the influence of mixture CMC 2 (lavender + eucalyptus + tea tree). Number (%) of specimens present at a distance greater than/equal to 3 cm from the source of the tested emulsion.

T (min)	CMC 2						Controls					
	500 µl/ 7 cm²			250 µl/ 7 cm²			500 µl/ 7 cm²			250 µl/ 7 cm²		
	Females N (%)	Males N (%)	Adults* N (%)	Females N (%)	Males N (%)	Adults N (%)	Females N (%)	Males N (%)	Adults N (%)	Females N (%)	Males N (%)	Adults N (%)
**15**	8 (100)	4 (100)	12 (100)	8 (100)	4 (100)	12 (100)	2 (25)**	1 (25)	3 (25)	2 (25)	0	2 (16.6)
**30**	8 (100)	4 (100)	12 (100)	8 (100)	4 (100)	12 (100)	1 (12.5)	0	1 (8.3)	1 (12.5)	0	1 (8.3)
**45**	8 (100)	4 (100)	12 (100)	8 (100)	4 (100)	12 (100)	2 (25)	0	2 (16.6)	0	0	0
**60**	8 (100)	4 (100)	12 (100)	8 (100)	4 (100)	12 (100)	1 (12.5)	0	1 (8.3)	1 (12.5)	0	1 (8.3)
**90**	8 (100)	4 (100)	12 (100)	8 (100)	4 (100)	12 (100)	2 (25)	0	2 (16.6)	1 (12.5)	0	1 (8.3)
**120**	8 (100)	4 (100)	12 (100)	8 (100)	4 (100)	12 (100)	2 (25)	0	2 (16.6)	0	0	0
**150**	8 (100)	4 (100)	12 (100)	8 (100)	4 (100)	12 (100)	3 (37.5)	0	3 (25)	1 (12.5)	0	1 (8.3)
**180**	8 (100)	4 (100)	12 (100)	8 (100)	4 (100)	12 (100)	2 (25)	0	2 (16.6)	1 (12.5)	1 (25)	2 (16.6)
**240**	8 (100)	3 (75)	11 (91.6)**	8 (100)	4 (100)	12 (100)	3 (37.5)	0	3 (25)	1 (12.5)	1 (25)	2 (16.6)

T – Time after application of the tested emulsion/H_2O, N- number of ticks, * in every experimental group, as in the single control, 12 adults of *D. reticulatus* (8 females and 4 males) were used, ** all other specimens not included in the table were located at the site of application of the tested emulsion/H_2O and/or at a distance less than 3 cm from the site of its application.

Table S3. Behavior of *Dermacentor reticulatus* adults under the influence of mixture CMC 3 (thyme + peppermint). Number (%) of specimens present at a distance greater than/equal to 3 cm from the source of the tested emulsion.

	**CMC 3**						**Controls**					
	500 µl/ 7 cm²			250 µl/ 7 cm²			500 µl/ 7 cm²			250 µl/ 7 cm²		
T (min)	Females N (%)	Males N (%)	Adults* N (%)	Females N (%)	Males N (%)	Adults N (%)	Females N (%)	Males N (%)	Adults N (%)	Females N (%)	Males N (%)	Adults N (%)
**15**	8 (100)	4 (100)	12 (100)	8 (100)	4 (100)	12 (100)	2 (25)	1 (25)	3 (25)	2 (25)	0	2 (16.6)
**30**	8 (100)	4 (100)	12 (100)	8 (100)	4 (100)	12 (100)	1 (12.5)	0	1 (8.3)	1 (12.5)	0	1 (8.3)
**45**	8 (100)	4 (100)	12 (100)	8 (100)	4 (100)	12 (100)	2 (25)	0	2 (16.6)	0	0	0
**60**	8 (100)	4 (100)	12 (100)	8 (100)	4 (100)	12 (100)	1 (12.5)	0	1 (8.3)	1 (12.5)	0	1 (8.3)
**90**	8 (100)	4 (100)	12 (100)	8 (100)	4 (100)	12 (100)	2 (25)	0	2 (16.6)	1 (12.5)	0	1 (8.3)
**120**	8 (100)	4 (100)	12 (100)	8 (100)	4 (100)	12 (100)	2 (25)	0	2 (16.6)	0	0	0
**150**	8 (100)	4 (100)	12 (100)	7 (87.5)	4 (100)	11 (91.6)	3 (37.5)	0	3 (25)	1 (12.5)	0	1 (8.3)
**180**	8 (100)	4 (100)	12 (100)	7 (87.5)	2 (50)	9 (75)	2 (25)	0	2 (16.6)	1 (12.5)	1 (25)	2 (16.6)
**240**	7 (87.5)	4 (100)	11 (91.6)**	6 (75)	2 (50)	8 (66.6)	3 (37.5)	0	3 (25)	1 (12.5)	1 (25)	2 (16.6)

T – Time after application of the tested emulsion/H_2O, N- number of ticks, * in every experimental group, as in the single control, 12 adults of *D. reticulatus* (8 females and 4 males) were used, ** all other specimens not included in the table were located at the site of application of the tested emulsion/H_2O and/or at a distance less than 3 cm from the site of its application.

Table S4. Behavior of *Dermacentor reticulatus* adults under the influence of mixture CMC 4 (vanilla + lavender). Number (%) of specimens present at a distance greater than/equal to 3 cm from the source of the tested emulsion.

T (min)	**CMC 4**						**Controls**					
	500 µl/ 7 cm²			250 µl/ 7 cm²			500 µl/ 7 cm²			250 µl/ 7 cm²		
	Females N (%)	Males N (%)	Adults* N (%)	Females N (%)	Males N (%)	Adults N (%)	Females N (%)	Males N (%)	Adults N (%)	Females N (%)	Males N (%)	Adults N (%)
**15**	8 (100)	4 (100)	12 (100)	8 (100)	4 (100)	12 (100)	2 (25)	1 (25)	3 (25)	2 (25)	0	2 (16.6)
**30**	8 (100)	4 (100)	12 (100)	8 (100)	4 (100)	12 (100)	1 (12.5)	0	1 (8.3)	1 (12.5)	0	1 (8.3)
**45**	8 (100)	4 (100)	12 (100)	8 (100)	4 (100)	12 (100)	2 (25)	0	2 (16.6)	0	0	0
**60**	8 (100)	4 (100)	12 (100)	7 (87.5)	4 (100)	11 (91.6)	1 (12.5)	0	1 (8.3)	1 (12.5)	0	1 (8.3)
**90**	8 (100)	4 (100)	12 (100)	7 (87.5)	3 (75)	10 (83.3)	2 (25)	0	2 (16.6)	1 (12.5)	0	1 (8.3)
**120**	7** (87.5)	4 (100)	11 (91.6)	6 (75)	4 (100)	10 (83.3)	2 (25)	0	2 (16.6)	0	0	0
**150**	5 (62.5)	2 (50)	7 (58.3)	7 (87.5)	4 (100)	11 (91.6)	3 (37.5)	0	3 (25)	1 (12.5)	0	1 (8.3)
**180**	5 (62.5)	3 (75)	8 (66.6)	5 (62.5)	3 (75)	8 (66.6)	2 (25)	0	2 (16.6)	1 (12.5)	1 (25)	2 (16.6)
**240**	5 (62.5)	3 (75)	8 (66.6)	6 (75)	3 (75)	9 (75)	3 (37.5)	0	3 (25)	1 (12.5)	1 (25)	2 (16.6)

T – Time after application of the tested emulsion/H_2O, N- number of ticks, * in every experimental group, as in the single control, 12 adults of *D. reticulatus* (8 females and 4 males) were used, ** all other specimens not included in the table were located at the site of application of the tested emulsion/H_2O and/or at a distance less than 3 cm from the site of its application.

Table S5. Behavior of *Dermacentor reticulatus* adults under the influence of mixture CMC 5 (cloves + patchouli). Number (%) of specimens present at a distance greater than/equal to 3 cm from the source of the tested emulsion.

	**CMC 5**						**Controls**					
	500 µl/ 7 cm^2			250 µl/ 7 cm^2			500 µl/ 7 cm^2			250 µl/ 7 cm^2		
T (min)	Females N (%)	Males N (%)	Adults* N (%)	Females N (%)	Males N (%)	Adults N (%)	Females N (%)	Males N (%)	Adults N (%)	Females N (%)	Males N (%)	Adults N (%)
**15**	4 (50)**	4 (100)	8 (66.6)	8 (100)	4 (100)	12 (100)	2 (25)	1 (25)	3 (25)	2 (25)	0	2 (16.6)
**30**	6 (75)	4 (100)	10 (83.3)	8 (100)	4 (100)	12 (100)	1 (12.5)	0	1 (8.3)	1 (12.5)	0	1 (8.3)
**45**	7 (87.5)	4 (100)	11 (91.6)	8 (100)	4 (100)	12 (100)	2 (25)	0	2 (16.6)	0	0	0
**60**	8 (100)	4 (100)	12 (100)	8 (100)	4 (100)	12 (100)	1 (12.5)	0	1 (8.3)	1 (12.5)	0	1 (8.3)
**90**	7 (87.5)	4 (100)	11 (91.6)	6 (75)	4 (100)	10 (83.3)	2 (25)	0	2 (16.6)	1 (12.5)	0	1 (8.3)
**120**	8 (100)	4 (100)	12 (100)	7 (87.5)	4 (100)	11 (91.6)	2 (25)	0	2 (16.6)	0	0	0
**150**	8 (100)	4 (100)	12 (100)	6 (75)	4 (100)	10 (83.3)	3 (37.5)	0	3 (25)	1 (12.5)	0	1 (8.3)
**180**	8 (100)	4 (100)	12 (100)	7 (87.5)	4 (100)	11 (91.6)	2 (25)	0	2 (16.6)	1 (12.5)	1 (25)	2 (16.6)
**240**	8 (100)	4 (100)	12 (100)	8 (100)	4 (100)	12 (100)	3 (37.5)	0	3 (25)	1 (12.5)	1 (25)	2 (16.6)

T – Time after application of the tested emulsion/H_2O, N- number of ticks, * in every experimental group, as in the single control, 12 adults of *D. reticulatus* (8 females and 4 males) were used, ** all other specimens not included in the table were located at the site of application of the tested emulsion/H_2O and/or at a distance less than 3 cm from the site of its application.

Table S6. Behavior of *Dermacentor reticulatus* adults under the influence of mixture CHIT 1 (citronella + rosemary + geranium). Number (%) of specimens present at a distance greater than/equal to 3 cm from the source of the tested emulsion.

T (min)	CHIT 1						Controls					
	500 µl/ 7 cm²			250 µl/ 7 cm²			500 µl/ 7 cm²			250 µl/ 7 cm²		
	Females N (%)	Males N (%)	Adults* N (%)	Females N (%)	Males N (%)	Adults N (%)	Females N (%)	Males N (%)	Adults N (%)	Females N (%)	Males N (%)	Adults N (%)
**15**	8 (100)	4 (100)	12 (100)	8 (100)	3 (75)	11 (91.6)	2 (25)	1 (25)	3 (25)	2 (25)	0	2 (16.6)
**30**	8 (100)	4 (100)	12 (100)	8 (100)	4 (100)	12 (100)	1 (12.5)	0	1 (8.3)	1 (12.5)	0	1 (8.3)
**45**	7 (87.5)**	3 (75)	10 (83.3)	8 (100)	4 (100)	12 (100)	2 (25)	0	2 (16.6)	0	0	0
**60**	8 (100)	4 (100)	12 (100)	8 (100)	4 (100)	12 (100)	1 (12.5)	0	1 (8.3)	1 (12.5)	0	1 (8.3)
**90**	6 (75)	3 (75)	9 (75)	8 (100)	4 (100)	12 (100)	2 (25)	0	2 (16.6)	1 (12.5)	0	1 (8.3)
**120**	7 (87.5)	2 (50)	9 (75)	6 (75)	3 (75)	9 (75)	2 (25)	0	2 (16.6)	0	0	0
**150**	4 (50)	2 (50)	6 (50)	5 (62.5)	4 (100)	9 (75)	3 (37.5)	0	3 (25)	1 (12.5)	0	1 (8.3)
**180**	3 (37.5)	3 (75)	6 (50)	4 (50)	4 (100)	8 (66.6)	2 (25)	0	2 (16.6)	1 (12.5)	1 (25)	2 (16.6)
**240**	6 (75)	3 (75)	9 (75)	4 (50)	4 (100)	8 (66.6)	3 (37.5)	0	3 (25)	1 (12.5)	1 (25)	2 (16.6)

T – Time after application of the tested emulsion/H_2O, N- number of ticks, * in every experimental group, as in the single control, 12 adults of *D. reticulatus* (8 females and 4 males) were used, ** all other specimens not included in the table were located at the site of application of the tested emulsion/H_2O and/or at a distance less than 3 cm from the site of its application.

Table S7. Behavior of *Dermacentor reticulatus* adults under the influence of mixture CHIT 2 (lavender + eucalyptus + tea tree). Number (%) of specimens present at a distance greater than/equal to 3 cm from the source of the tested emulsion.

T (min)	**CHIT 2**						**Controls**					
	500 µl/ 7 cm²			250 µl/ 7 cm²			500 µl/ 7 cm²			250 µl/ 7 cm²		
	Females N (%)	Males N (%)	Adults* N (%)	Females N (%)	Males N (%)	Adults N (%)	Females N (%)	Males N (%)	Adults N (%)	Females N (%)	Males N (%)	Adults N (%)
**15**	8 (100)	4 (100)	12 (100)	8 (100)	4 (100)	12 (100)	2 (25)	1 (25)	3 (25)	2 (25)	0	2 (16.6)
**30**	8 (100)	4 (100)	12 (100)	8 (100)	4 (100)	12 (100)	1 (12.5)	0	1 (8.3)	1 (12.5)	0	1 (8.3)
**45**	8 (100)	4 (100)	12 (100)	8 (100)	4 (100)	12 (100)	2 (25)	0	2 (16.6)	0	0	0
**60**	8 (100)	4 (100)	12 (100)	8 (100)	4 (100)	12 (100)	1 (12.5)	0	1 (8.3)	1 (12.5)	0	1 (8.3)
**90**	8 (100)	4 (100)	12 (100)	8 (100)	4 (100)	12 (100)	2 (25)	0	2 (16.6)	1 (12.5)	0	1 (8.3)
**120**	8 (100)	4 (100)	12 (100)	7 (87.5)	3 (75)	10 (83.3)	2 (25)	0	2 (16.6)	0	0	0
**150**	8 (100)	4 (100)	12 (100)	6 (75)	4 (100)	10 (83.3)	3 (37.5)	0	3 (25)	1 (12.5)	0	1 (8.3)
**180**	7 (87.5)**	4 (100)	11 (91.6)	7 (87.5)	4 (100)	11 (91.6)	2 (25)	0	2 (16.6)	1 (12.5)	1 (25)	2 (16.6)
**240**	8 (100)	4 (100)	12 (100)	8 (100)	4 (100)	12 (100)	3 (37.5)	0	3 (25)	1 (12.5)	1 (25)	2 (16.6)

T – Time after application of the tested emulsion/H_2O, N- number of ticks, * in every experimental group, as in the single control, 12 adults of *D. reticulatus* (8 females and 4 males) were used, ** all other specimens not included in the table were located at the site of application of the tested emulsion/H_2O and/or at a distance less than 3 cm from the site of its application.

Table S8. Behavior of *Dermacentor reticulatus* adults under the influence of mixture CHIT 3 (thyme + peppermint). Number (%) of specimens present at a distance greater than/equal to 3 cm from the source of the tested emulsion.

T (min)	**CHIT 3**						**Controls**					
	500 µl/ 7 cm²			250 µl/ 7 cm²			500 µl/ 7 cm²			250 µl/ 7 cm²		
	Females N (%)	Males N (%)	Adults* N (%)	Females N (%)	Males N (%)	Adults N (%)	Females N (%)	Males N (%)	Adults N (%)	Females N (%)	Males N (%)	Adults N (%)
**15**	6 (75)**	3 (75)	9 (75)	8 (100)	4 (100)	12 (100)	2 (25)	1 (25)	3 (25)	2 (25)	0	2 (16.6)
**30**	7 (87.5)	4 (100)	11 (91.6)	8 (100)	3 (75)	11 (91.6)	1 (12.5)	0	1 (8.3)	1 (12.5)	0	1 (8.3)
**45**	6 (75)	3 (75)	9 (75)	8 (100)	4 (100)	12 (100)	2 (25)	0	2 (16.6)	0	0	0
**60**	8 (100)	3 (75)	11 (91.6)	8 (100)	4 (100)	12 (100)	1 (12.5)	0	1 (8.3)	1 (12.5)	0	1 (8.3)
**90**	6 (75)	3 (75)	9 (75)	8 (100)	3 (75)	11 (91.6)	2 (25)	0	2 (16.6)	1 (12.5)	0	1 (8.3)
**120**	7 (87.5)	3 (75)	10 (83.3)	8 (100)	3 (75)	11 (91.6)	2 (25)	0	2 (16.6)	0	0	0
**150**	8 (100)	2 (50)	10 (83.3)	7 (87.5)	4 (100)	11 (91.6)	3 (37.5)	0	3 (25)	1 (12.5)	0	1 (8.3)
**180**	7 (87.5)	3 (75)	10 (83.3)	7 (87.5)	3 (75)	10 (83.3)	2 (25)	0	2 (16.6)	1 (12.5)	1 (25)	2 (16.6)
**240**	7 (87.5)	4 (100)	11 (91.6)	8 (100)	4 (100)	12 (100)	3 (37.5)	0	3 (25)	1 (12.5)	1 (25)	2 (16.6)

T – Time after application of the tested emulsion/H_2O, N- number of ticks, * in every experimental group, as in the single control, 12 adults of *D. reticulatus* (8 females and 4 males) were used, ** all other specimens not included in the table were located at the site of application of the tested emulsion/H_2O and/or at a distance less than 3 cm from the site of its application.

Table S9. Behavior of *Dermacentor reticulatus* adults under the influence of mixture CHIT 4 (vanilla + lavender). Number (%) of specimens present at a distance greater than/equal to 3 cm from the source of the tested emulsion.

T (min)	CHIT 4						Controls					
	500 μl/ 7 cm²			250 μl/ 7 cm²			500 μl/ 7 cm²			250 μl/ 7 cm²		
	Females N (%)	Males N (%)	Adults* N (%)	Females N (%)	Males N (%)	Adults N (%)	Females N (%)	Males N (%)	Adults N (%)	Females N (%)	Males N (%)	Adults N (%)
**15**	8 (100)	4 (100)	12 (100)	8 (100)	4 (100)	12 (100)	2 (25)	1 (25)	3 (25)	2 (25)	0	2 (16.6)
**30**	8 (100)	4 (100)	12 (100)	8 (100)	4 (100)	12 (100)	1 (12.5)	0	1 (8.3)	1 (12.5)	0	1 (8.3)
**45**	8 (100)	4 (100)	12 (100)	8 (100)	4 (100)	12 (100)	2 (25)	0	2 (16.6)	0	0	0
**60**	8 (100)	4 (100)	12 (100)	8 (100)	4 (100)	12 (100)	1 (12.5)	0	1 (8.3)	1 (12.5)	0	1 (8.3)
**90**	8 (100)	4 (100)	12 (100)	8 (100)	4 (100)	12 (100)	2 (25)	0	2 (16.6)	1 (12.5)	0	1 (8.3)
**120**	8 (100)	4 (100)	12 (100)	8 (100)	4 (100)	12 (100)	2 (25)	0	2 (16.6)	0	0	0
**150**	7 (87.5)**	4 (100)	11 (91.6)	8 (100)	3 (75)	11 (91.6)	3 (37.5)	0	3 (25)	1 (12.5)	0	1 (8.3)
**180**	6 (75)	4 (100)	10 (83.3)	7 (87.5)	3 (75)	10 (83.3)	2 (25)	0	2 (16.6)	1 (12.5)	1 (25)	2 (16.6)
**240**	5 (62.5)	3 (75)	8 (66.6)	8 (100)	3 (75)	11 (91.6)	3 (37.5)	0	3 (25)	1 (12.5)	1 (25)	2 (16.6)

T – Time after application of the tested emulsion/H_2O, N- number of ticks, * in every experimental group, as in the single control, 12 adults of *D. reticulatus* (8 females and 4 males) were used, ** all other specimens not included in the table were located at the site of application of the tested emulsion/H_2O and/or at a distance less than 3 cm from the site of its application.

Table S10. Behavior of *Dermacentor reticulatus* adults under the influence of mixture CHIT (cloves + patchouli). Number (%) of specimens present at a distance greater than/equal to 3 cm from the source of the tested emulsion.

T (min)	CHIT 5						Controls					
	500 µl/ 7 cm^2			250 µl/ 7 cm^2			500 µl/ 7 cm^2			250 µl/ 7 cm^2		
	Females N (%)	Males N (%)	Adults* N (%)	Females N (%)	Males N (%)	Adults N (%)	Females N (%)	Males N (%)	Adults* N (%)	Females N (%)	Males N (%)	Adults N (%)
**15**	5 (62.5)**	4 (100)	9 (75)	5 (62.5)	4 (100)	9 (75)	2 (25)	1 (25)	3 (25)	2 (25)	0	2 (16.6)
**30**	7 (87.5)	4 (100)	11 (91.6)	6 (75)	4 (100)	10 (83.3)	1 (12.5)	0	1 (8.3)	1 (12.5)	0	1 (8.3)
**45**	7 (87.5)	4 (100)	11 (91.6)	7 (87.5)	4 (100)	11 (91.6)	2 (25)	0	2 (16.6)	0	0	0
**60**	8 (100)	4 (100)	12 (100)	6 (75)	4 (100)	10 (83.3)	1 (12.5)	0	1 (8.3)	1 (12.5)	0	1 (8.3)
**90**	7 (87.5)	4 (100)	11 (91.6)	6 (75)	4 (100)	10 (83.3)	2 (25)	0	2 (16.6)	1 (12.5)	0	1 (8.3)
**120**	7 (87.5)	4 (100)	11 (91.6)	6 (75)	3 (75)	9 (75)	2 (25)	0	2 (16.6)	0	0	0
**150**	8 (100)	4 (100)	12 (100)	7 (87.5)	3 (75)	10 (83.3)	3 (37.5)	0	3 (25)	1 (12.5)	0	1 (8.3)
**180**	8 (100)	4 (100)	12 (100)	7 (87.5)	4 (100)	11 (91.6)	2 (25)	0	2 (16.6)	1 (12.5)	1 (25)	2 (16.6)
**240**	8 (100)	2 (50)	10 (83.3)	7 (87.5)	4 (100)	11 (91.6)	3 (37.5)	0	3 (25)	1 (12.5)	1 (25)	2 (16.6)

T – Time after application of the tested emulsion/H_2O, N- number of ticks, * in every experimental group, as in the single control, 12 adults of *D. reticulatus* (8 females and 4 males) were used, ** all other specimens not included in the table were located at the site of application of the tested emulsion/H_2O and/or at a distance less than 3 cm from the site of its application.

Table S11. Behavior of *Ixodes ricinus* females under the influence of selected emulsions. Number (%) of specimens present at a distance greater than/equal to 3 cm from the source of the tested emulsion.

T (min)	250 µl/ 7 cm²				Control
	**CMC 2**	**CMC 3**	**CHIT 2**	**CHIT 4**	
	Females N* (%)	Females N (%)	Females N (%)	Females N (%)	Females N (%)
**15**	16 (100)	16 (100)	16 (100)	15** (93.7)	11 (68.7)
**30**	15 (93.75)**	16 (100)	16 (100)	14 (87.5)	13 (81.2)
**45**	16 (100)	16 (100)	16 (100)	16 (100)	12 (75)
**60**	16 (100)	16 (100)	16 (100)	16 (100)	11 (68.7)
**90**	16 (100)	16 (100)	16 (100)	14 (87.5)	11 (68.7)
**120**	16 (100)	15 (93.7)*	15 (93.7)	15 (93.7)	8 (50)
**150**	15 (93.75)	14 (87.5)	16 (100)	13 (81.2)	10 (62.5)
**180**	16 (100)	15 (93.7)	13 (81.2)	16 (100)	10 (62.5)
**240**	16 (100)	15 (93.7)	16 (100)	14 (87.5)	9 (56.2)

T - Time after application of the tested emulsion/H_2O, N- number of ticks, *in the test groups, as in the control, 16 females of *I. ricinus* were used, ** all other specimens not included in the table were located at the site of application of the tested emulsion/H_2O and/or at a distance less than 3 cm from the site of its application, CMC- sodium carboxymethyl cellulose; CHIT- chitosan lactate.

Table S12. Behavior of *Dermacentor reticulatus* adults under the influence of sodium carboxymethyl cellulose (CMC). Number (%) of specimens present at a distance greater than/equal to 3 cm from the source of the tested emulsion.

T (min)	CMC						Controls					
	500 µl/ 7 cm^2			250 µl/ 7 cm^2			500 µl/ 7 cm^2			250 µl/ 7 cm^2		
	Females N (%)	Males N (%)	Adults* N (%)	Females N (%)	Males N (%)	Adults N (%)	Females N (%)	Males N (%)	Adults* N (%)	Females N (%)	Males N (%)	Adults N (%)
**15**	5 (62,5)*	1 (25)	6 (50)	8 (100)*	3 (75)	11 (91,6)	5 (62.5)	2 (50)	7 (58.3)	6 (75)*	2 (50)	8 (66.6)
**30**	3 (37,5)	1 (25)	4 (33.3)	6 (75)	4 (100)	10 (83,3)	3 (37.5)	3 (75)	6 (50)	3 (37.5)	3 (75)	6 (50)
**45**	6 (75)	1 (25)	7 (58.3)	6 (75)	4 (100)	10 (83.3)	3 (37.5)	3 (75)	6 (50)	6 (75)	2 (50)	8 (66.6)
**60**	5 (62.5)	1 (25)	6 (50)	5 (62.5)	4 (100)	9 (75)	3 (37.5)	2 (50)	5 (41.6)	5 (62.5)	4 (100)	9 (75)
**90**	5 (62.5)	2 (50)	7 (58.3)	4 (50)	4 (100)	8 (66.6)	2 (25)	2 (50)	4 (33.3)	4 (50)	2 (50)	6 (50)
**120**	3 (37.5)	2 (50)	5 (41.6)	2 (25)	4 (100)	6 (50)	4 (50)	2 (50)	6 (50)	4 (50)	4 (100)	8 (66.6)
**150**	3 (37.5)	2 (50)	5 (41.6)	4 (50)	2 (50)	6 (50)	1 (12.5)	3 (75)	4 (33.3)	2 (25)	1 (25)	3 (25)
**180**	3 (37.5)	2 (50)	5 (41.6)	4 (50)	4 (100)	8 (66.6)	3 (37.5)	4 (100)	7 (58.3)	3 (37.5)	0	3 (25)
**240**	3 (37.5)	2 (50)	5 (41.6)	2 (25)	3 (75)	5 (41.6)	3 (37.5)	4 (100)	7 (58.3)	4 (50)	2 (50)	6 (50)

T – Time after application of the tested matrix/H_2O, N- number of ticks, * in every experimental group, as in the single control, 12 adults of *D. reticulatus* (8 females and 4 males) were used, ** all other specimens not included in the table were located at the site of application of the tested matrix/H_2O and/or at a distance less than 3 cm from the site of its application.

Table S13. Behavior of *Dermacentor reticulatus* adults under the influence of chitosan lactate (CHIT). Number (%) of specimens present at a distance greater than/equal to 3 cm from the source of the tested emulsion.

T (min)	CHIT						Controls					
	500 µl/ 7 cm²			250 µl/ 7 cm²			500 µl/ 7 cm²			250 µl/ 7 cm²		
	Females N (%)	Males N (%)	Adults* N (%)	Females N (%)	Males N (%)	Adults N (%)	Females N (%)	Males N (%)	Adults* N (%)	Females N (%)	Males N (%)	Adults N (%)
**15**	4 (50)**	2 (50)	6 (50)	5 (62.5)**	3 (75)	8 (66.6)	5 (62.5)	2 (50)	7 (58.3)	6 (75)*	2 (50)	8 (66.6)
**30**	4 (50)	2 (50)	6 (50)	3 (37.5)	1 (25)	4 (33.3)	3 (37.5)	3 (75)	6 (50)	3 (37.5)	3 (75)	6 (50)
**45**	4 (50)	2 (50)	6 (50)	4 (50)	2 (50)	6 (50)	3 (37.5)	3 (75)	6 (50)	6 (75)	2 (50)	8 (66.6)
**60**	5 (62.5)	3 (75)	8 (66.6)	4 (50)	3 (75)	7 (58.3)	3 (37.5)	2 (50)	5 (41.6)	5 (62.5)	4 (100)	9 (75)
**90**	5 (62.5)	3 (75)	8 (66.6)	3 (37.5)	3 (75)	6 (50)	2 (25)	2 (50)	4 (33.3)	4 (50)	2 (50)	6 (50)
**120**	4 (50)	3 (75)	7 (58.3)	1 (12.5)	1 (25)	2 (16.6)	4 (50)	2 (50)	6 (50)	4 (50)	4 (100)	8 (66.6)
**150**	4 (50)	3 (75)	7 (58.3)	1 (12.5)	2 (50)	3 (25)	1 (12.5)	3 (75)	4 (33.3)	2 (25)	1 (25)	3 (25)
**180**	4 (50)	3 (75)	7 (58.3)	2 (25)	3 (75)	5 (41.6)	3 (37.5)	4 (100)	7 (58.3)	3 (37.5)	0	3 (25)
**240**	4 (50)	3 (75)	7 (58.3)	0	3 (75)	3 (25)	3 (37.5)	4 (100)	7 (58.3)	4 (50)	2 (50)	6 (50)

T- Time after application of the tested matrix/H_2O, N- number of ticks, * in every experimental group, as in the single control, 12 adults of *D. reticulatus* (8 females and 4 males) were used, ** all other specimens not included in the table were located at the site of application of the tested matrix/H_2O and/or at a distance less than 3 cm from the site of its application.
